# Chemical Composition and Anti-Lung Cancer Activities of *Melaleuca quinquenervia* Leaf Essential Oil: Integrating Gas Chromatography–Mass Spectrometry (GC/MS) Profiling, Network Pharmacology, and Molecular Docking

**DOI:** 10.3390/ph18060771

**Published:** 2025-05-22

**Authors:** Eman Fikry, Raha Orfali, Shagufta Perveen, Safina Ghaffar, Azza M. El-Shafae, Maher M. El-Domiaty, Nora Tawfeek

**Affiliations:** 1 Department of Pharmacognosy, Faculty of Pharmacy, Zagazig University, Zagazig 44519, Egypt; efhassan@zu.edu.eg (E.F.); amelshafaey@pharmacy.zu.edu.eg (A.M.E.-S.); mmeldomyaty@pharmacy.zu.edu.eg (M.M.E.-D.); noratawfeek@zu.edu.eg (N.T.); 2 Department of Pharmacognosy, Collage of Pharmacy, King Saud University, P.O. Box 2457, Riyadh 11451, Saudi Arabia; sghafar.c@ksu.edu.sa; 3 Department of Bacteriology, University of Wisconsin-Madison, Madison, WI 53706, USA

**Keywords:** *Melaleuca quinquenervia*, essential oil, lung cancer, cytotoxicity, in silico analysis, multi-target therapy

## Abstract

**Background/Objectives:** This study investigates the phytochemical composition and anticancer activity of *Melaleuca quinquenervia* leaf essential oil (MQLEO) from Egypt. **Methods:** Chemical profiling was performed using GC/MS. Anticancer activity was assessed through cytotoxicity screening against multiple cancer cell lines, with a subsequent evaluation of cell migration, apoptosis, and cell cycle analysis on the most sensitive line (A549). Network pharmacology and molecular docking analyses were employed to identify potential molecular targets and pathways. **Results:** GC/MS analysis revealed a unique profile dominated by 1,8-cineole (31.57%), α-pinene isomers (both 1R and 1S forms, collectively 21.26%), and sesquiterpene alcohols (viridiflorol: 13.65%; ledol: 4.55%). These results diverge from prior studies, showing a 25.63% decrease in 1,8-cineole and no detectable α-terpineol, suggesting environmental, genetic, or methodological impacts on biosynthesis. In vitro tests revealed selective cytotoxicity against A549 lung cancer cells (IC_50_ = 18.09 μg/mL; selectivity index = 4.30), meeting NCI criteria. Staurosporine was used as a positive control to validate the assays, confirming the reliability of the methods. MQLEO also inhibited cell migration (62–68% wound closure reduction) and induced apoptosis (24.32% vs. 0.7% in controls). Cell cycle arrest at the G_0_-G_1_ phase implicated cyclin-dependent kinase regulation. Network pharmacology identified ESR1, CASP3, PPARG, and PTGS2 as key targets, with MQLEO components engaging apoptosis, inflammation (TNF, IL-17), and estrogen pathways. **Conclusions:** MQLEO demonstrates promising anticancer activity through multiple mechanisms including apoptosis induction, cell cycle arrest, and migration inhibition. The multi-target activity profile highlights its potential as a therapeutic candidate for lung cancer, warranting further in vivo validation and pharmacokinetic studies to advance clinical translation.

## 1. Introduction

Cancer is a multifaceted disease defined by uncontrolled cell growth and the ability to invade surrounding tissues or metastasize to distant sites, primarily due to the disruption of normal cellular regulatory pathways. Despite substantial progress in therapeutic modalities, cancer continues to be a leading cause of death and disability globally, underscoring the urgent need for novel and more effective treatment strategies [1]. Conventional chemotherapy, though effective, often suffers from a lack of specificity, leading to collateral damage in healthy tissues and resulting in adverse effects such as bone marrow suppression, gastrointestinal toxicity, and hair loss. In contrast, natural products tend to exhibit reduced toxicity and act on multiple molecular targets simultaneously, thereby offering a potentially safer and more effective therapeutic approach [2].

Modern anticancer approaches often aim to disrupt the tumor’s capacity for repair and progression by targeting processes such as cell migration, angiogenesis, and inflammatory responses. For instance, agents like paclitaxel and vinblastine interfere with microtubule formation to hinder cellular movement [3], while drugs such as bevacizumab inhibit vascular endothelial growth factor, thereby restricting neovascularization critical to tumor survival and expansion [4]. Moreover, numerous chemotherapeutic agents exert their effects by promoting apoptosis via intrinsic and extrinsic pathways or by halting cell cycle progression through cyclin-dependent kinase inhibition, effectively suppressing cell proliferation [5,6,7,8].

In recent years, essential oils have emerged as promising candidates in the search for anticancer agents. Owing to their complex chemical composition, they demonstrate notable antioxidant and immunomodulatory properties and have shown potential in combating multidrug resistance in cancer cells [9].

The Myrtaceae family, comprising approximately 132 genera and 5950 species, is renowned for its aromatic properties and rich essential oil content [10,11]. Among its members, the genus *Melaleuca*, which includes nearly 382 species, known as paperbarks, honey-myrtles, bottlebrushes, or tea-trees, primarily distributed in Australia, has been extensively studied for its diverse biological activities, including antimicrobial [12,13], anti-inflammatory [14,15], and cytotoxic effects [16,17,18,19,20]

*M. quinquenervia* (Cav.) S.T. Blake (syn. *M. viridiflora* var. *rubriflora* Pancher ex Brongn. & Gris) [21] is distinguished by its papery bark, aromatic evergreen leaves, and characteristic bottlebrush-like inflorescences. Its essential oil, particularly rich in 1,8-cineole, is traditionally used for respiratory and inflammatory ailments [11].

While the essential oil (EO) of *M. quinquenervia* leaves (MQLEO) has been analyzed in multiple regions, including Australia, Papua New Guinea [22], Vietnam [12], and Costa Rica [23], with a 1991 Egyptian study identifying 1,8-cineole (57.2%) as its major component [24], EO profiles are inherently influenced by geographic, climatic, and methodological variables [25,26]. Furthermore, modern analytical advancements now enable more precise and holistic phytochemical characterization [27,28]. Despite documented bioactivities such as anti-inflammatory [12], anti-tyrosinase, anti-melanogenic [29], antimicrobial, and enzyme inhibitory effects [12], no studies have investigated its antiproliferative properties.

**Figure 1:**
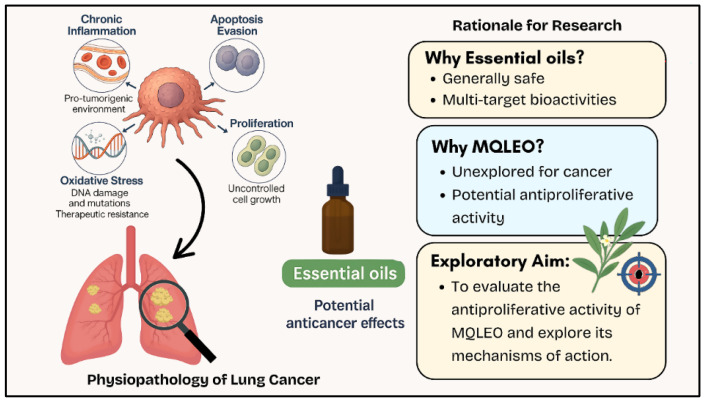
Illustrative representation of the key pathophysiological features of lung cancer and the rationale for investigating *Melaleuca quinquenervia* essential oil (MQLEO) as a potential anticancer agent.

To fill this knowledge gap, the present study investigates the cytotoxic effects of MQLEO on three human carcinoma cell lines: MCF-7 (breast), HepG2 (liver), and A549 (lung). These cell lines were selected to represent diverse tissues and tumorigenic mechanisms, facilitating a comprehensive assessment of MQLEO’s anticancer efficacy. Notably, lung cancer remains one of the most prevalent and fatal cancer types worldwide and is characterized by a complex interplay of pathological features, including chronic inflammation [30], resistance to apoptosis [31], oxidative damage [32], and metastasis [32]. These processes offer numerous molecular targets suitable for intervention with multi-target agents like essential oils (Figure 1).

Therefore, this study seeks to re-characterize the chemical composition of Egyptian MQLEO using modern GC-MS techniques and assess its antiproliferative potential through integrated in vitro and computational approaches (network pharmacology and molecular docking) to uncover mechanistic pathways. By addressing these gaps, this work advances the understanding MQLEO’s pharmacological versatility under region-specific conditions.

## 2. Results

### 2.1. Chemical Composition of MQLEO

**Figure 2:**
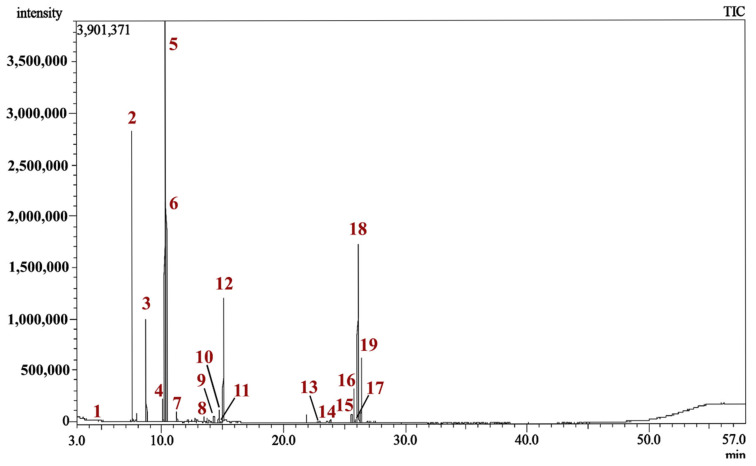
GC/MS chromatogram of *M. quinquenervia* leaf essential oil. Numbers indicate peaks identified in Table 1. The chemical structures of all identified compounds are provided in Supplementary Figure S1.

The Gas Chromatography–Mass Spectrometry (GC/MS) analysis of MQLEO led to the identification of 19 distinct chemical constituents, collectively representing 98.09% of its total composition. The relative abundance of each component, expressed as area percentages, is systematically outlined in Table 1. A representative chromatographic profile of MQLEO, obtained through GC/MS, is depicted in Figure 2. Additionally, the structural configurations of the detected constituents are provided in Supplementary Figure S1.

### 2.2. In Vitro Assessments

#### 2.2.1. Cytotoxic Potential and Selectivity of MQLEO

The cytotoxic potential of MQLEO was evaluated against VERO (Normal African Green Monkey Kidney Cells), MCF-7, HepG-2, and A-549 cancer cell lines using CC_50_ and IC_50_ values (Table 2). MQLEO exhibited a CC_50_ of 77.76 ± 3.96 μg/mL on VERO cells, indicating lower toxicity to normal cells compared to Staurosporine (CC_50_ = 24.20 ± 1.23 μg/mL). For cancer cell lines, MQLEO demonstrated IC_50_ values of 27.74 ± 1.41 μg/mL (MCF-7), 66.04 ± 3.36 μg/mL (HepG-2), and 18.09 ± 0.92 μg/mL (A-549). These values align with the National Cancer Institute (NCI) activity criterion (IC_50_ ≤ 30 μg/mL) for MCF-7 and A-549 but not for HepG-2 [43].

The SI, calculated as CC_50_/IC_50_, revealed significant selectivity (SI > 3) only for A-549 (SI = 4.30). For MCF-7, the SI was 2.80, falling below the critical threshold of 3, while HepG-2 showed minimal selectivity (SI = 1.18). Therefore, A549 was selected for further antiproliferative studies due to having the highest selectivity index and lowest IC_50_ value among the tested cancer cell lines, indicating both the superior efficacy and selectivity of MQLEO against this particular cell line.

To investigate the potential mechanisms behind MQLEO’s anticancer activity, a wound healing assay was conducted to evaluate its effects on cellular migration, a critical process in tumor metastasis.

#### 2.2.2. MQLEO-Mediated Suppression of Wound Healing and Metastatic Migration in A549 Lung Cancer Cells

**Figure 3:**
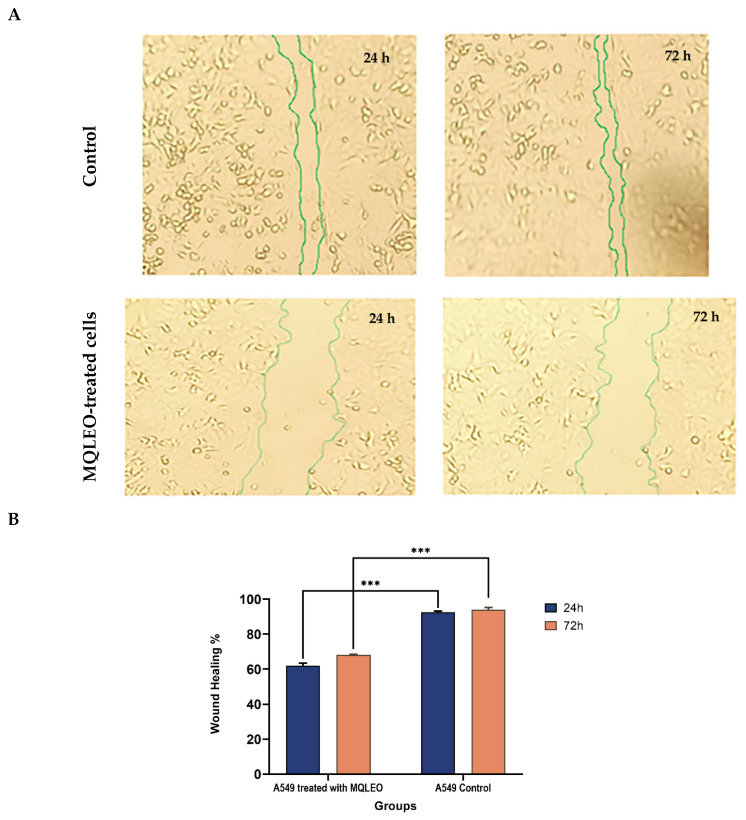
Wound healing assay in A549 cells. (**A**) Wound closure images of untreated and MQLEO-treated cells at 0, 24, and 72 h. (**B**) Quantified wound closure percentages (*n* = 3; mean ± SD). *** *p* < 0.001 vs. control.

The anti-migratory potential of MQLEO was evaluated using a wound healing assay on A549 lung adenocarcinoma cells over 24 and 72 h. MQLEO significantly suppressed wound closure compared to untreated controls (Figure 3). At 24 h post-treatment, wound healing in MQLEO-treated cells was reduced to approximately 62% of the untreated control, with further suppression observed at 72 h (68% closure). In contrast, untreated cells exhibited near-complete wound closure within the same timeframe. This marked inhibition of cell migration underscores MQLEO’s ability to interfere with cellular regeneration and extracellular matrix remodeling, key processes driving tumor cell metastasis.

The observed inhibition of A549 cell migration prompted further investigation into the molecular basis of MQLEO’s anticancer properties, specifically focusing on its potential to induce apoptosis and alter cell cycle distribution.

#### 2.2.3. Apoptosis and Cell Cycle Analysis of A549 Cells in Response to MQLEO

##### Apoptosis Induction by MQLEO

**Figure 4:**
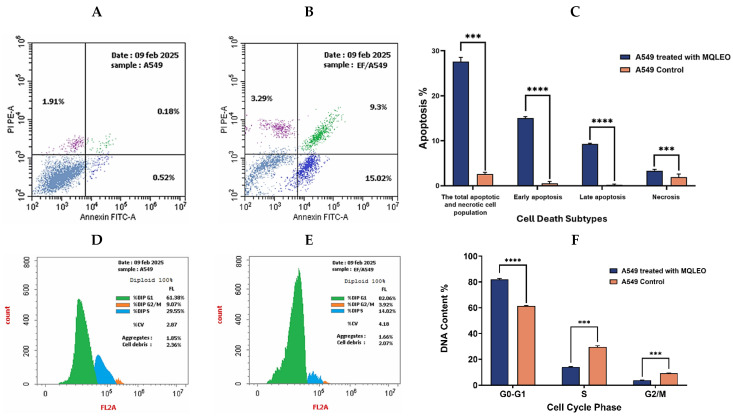
Apoptosis and cell cycle analysis in A549 cells. (**A**,**B**) Apoptosis profiles for untreated and MQLEO-treated cells. (**C**) Quantitative apoptosis data (*n* = 3; mean ± SD). (**D**,**E**) Cell cycle histograms for untreated and treated cells. (**F**) Cell cycle phase quantification (*n* = 3; mean ± SD). *** *p* < 0.001, **** *p* < 0.0001 vs. control.

The pro-apoptotic effect of MQLEO on A549 cells was evaluated using Annexin V/propidium iodide (PI) dual staining, followed by flow cytometry. As shown in Figure 4, MQLEO treatment induced a significant shift in the apoptotic and necrotic profiles of A549 cells compared to untreated controls. Quantitatively, MQLEO-exposed cells exhibited 15.02% early apoptosis (Annexin V^+^/PI^−^), 9.3% late apoptosis (Annexin V^+^/PI^+^), and 3.29% necrosis (Annexin V^−^/PI^+^). In contrast, control cells demonstrated minimal apoptosis and necrosis, with 0.52% early apoptosis, 0.18% late apoptosis, and 1.91% necrosis. The total apoptotic and necrotic cell population (early + late apoptosis + necrosis) increased from 2.61% in controls to 27.61% in MQLEO-treated cells, reflecting a 10.6-fold induction of total cell death.

##### MQLEO-Induced Cell Cycle Arrest

To further elucidate the mechanism of MQLEO’s antiproliferative action, cell cycle analysis was performed using PI staining to quantify DNA content. Following treatment with MQLEO at its IC_25_ concentration, a significant accumulation of cells was observed in the G_0_/G_1_ phase (Figure 4), suggesting cell cycle arrest at this checkpoint. No notable changes were detected in the S or G_2_/M phases, indicating that MQLEO specifically hinders the transition from G_1_ to S phase, thereby limiting DNA synthesis and subsequent cell division.

These findings collectively indicate that MQLEO exerts its antiproliferative effects on A549 cells by inducing programmed cell death and halting cell cycle progression at the G_0_/G_1_ phase.

### 2.3. Network Pharmacology-Guided Mechanistic Analysis

#### 2.3.1. Drug-likeness Profiling of Bioactive Constituents and Target Exploration

Pharmacokinetic evaluation serves as an integral component of drug development, facilitating the rational identification of candidate molecules with enhanced therapeutic efficacy, safety, and druggability [44]. As evidenced in Table S1, all 19 MQLEO-derived bioactive compounds demonstrated adherence to Lipinski’s rule of five parameters [36], each compound exhibited an Abbott oral bioavailability score ≥ 0.5, underscoring their potential for favorable gastrointestinal absorption and systemic bioavailability.

**Figure 5:**
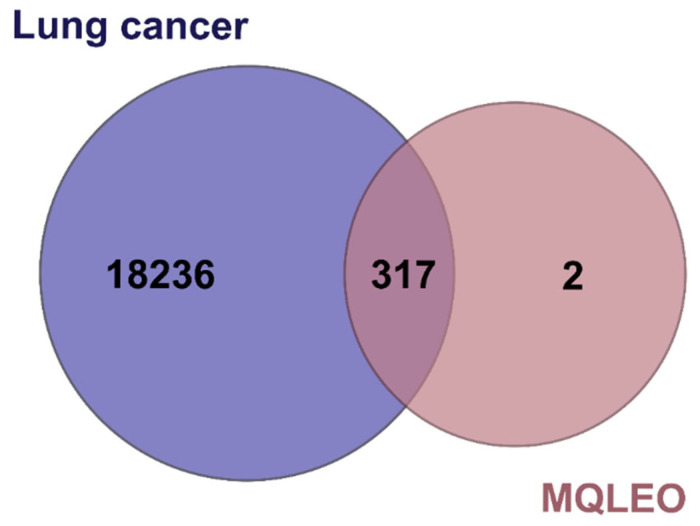
Overlapping molecular targets between lung cancer and MQLEO bioactive compounds. MQLEO, *Melaleuca quinquenervia* leaf essential oil.

To delineate the protein targets of MQLEO’s bioactive constituents, in silico target predictions were performed via Swiss Target Prediction and Target Net databases, generating 319 distinct molecular targets (Table S2). Concurrently, lung cancer-associated targets (*n* = 18,553; Table S3) were curated from OMIM, GeneCards, and DisGeNeT repositories. The intersectional mapping of compound- and disease-related targets identified 317 overlapping candidates (Table S4; Figure 5), highlighting potential therapeutic intersections between MQLEO bioactive and lung cancer pathogenesis.

#### 2.3.2. Identification and Topological Characterization of Hub Genes via Protein–Protein Interaction (PPI) Network Analysis

**Figure 6:**
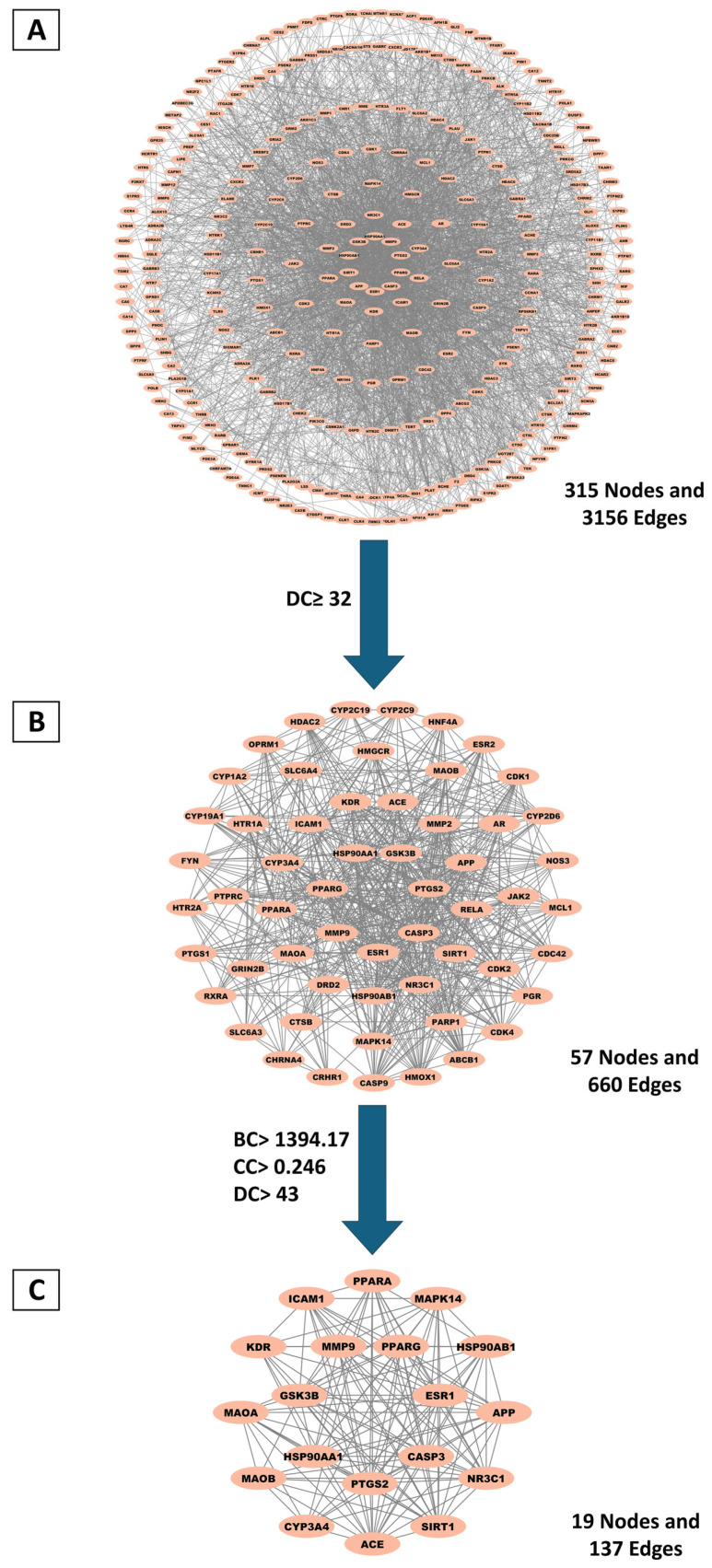
Key targets identified through PPI network analysis. (**A**) PPI network of 315 shared targets. (**B**) Initial selection using a threshold of twice the median degree centrality. (**C**) Refined targets based on betweenness, closeness, and degree centrality values exceeding median thresholds.

To investigate the therapeutic mechanism of MQLEO in lung cancer, overlapping targets were imported into the STRING database to construct a PPI network. Disconnected nodes were removed to optimize network reliability at a confidence threshold of 0.4, resulting in an initial network containing 315 nodes and 3156 edges (Figure 6A). Subsequent topological evaluation was conducted using Cytoscape 3.10.2, where node significance was assessed via the CytoNCA plugin by calculating centrality parameters: betweenness centrality (BC); closeness centrality (CC); degree centrality (DC) (Table S5).

Two sequential filtrations were performed to identify hub genes. The first selection applied a DC threshold (≥32, twice the median value), refining the network to 57 nodes and 660 edges (Figure 6B; Table S6). A subsequent stringent filtration, using median-based thresholds (BC ≥ 1394.17, CC ≥ 0.246, DC ≥ 43), further reduced the network to 19 core nodes and 137 edges (Figure 6C). These targets were ranked by DC, with detailed topological metrics provided in Table 3. Notably, ESR1, CASP3, PPARG, and PTGS2 were identified as the top-ranking candidates, suggesting their pivotal roles in the network. This multi-step topological analysis highlights critical targets potentially mediating MQLEO’s anticancer effects in lung cancer pathogenesis.

#### 2.3.3. Key MQLEO Bioactive Constituents Linked to Lung Cancer Targets

**Figure 7:**
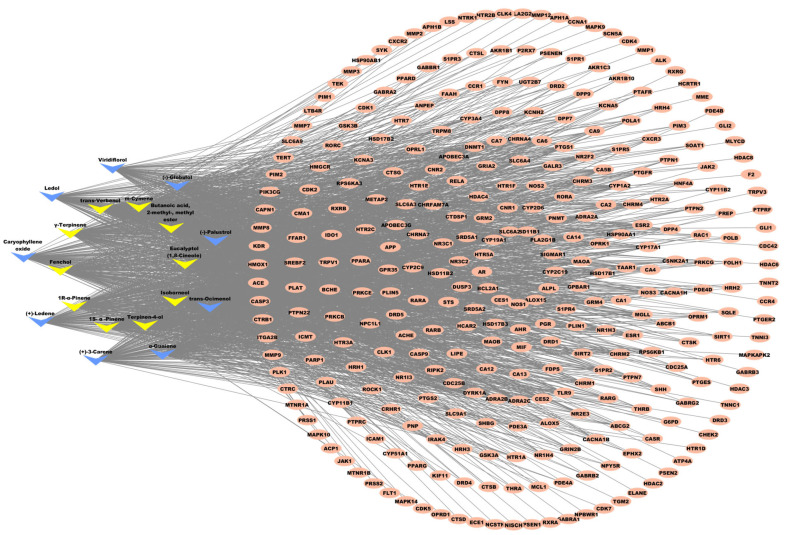
Interaction network between *M. quinquenervia* oil compounds and target proteins. Yellow arrows: high-degree compounds (DC ≥ 133; blue arrows: low-degree compounds; orange ovals: therapeutic targets.

A systematic computational investigation was performed to explore potential associations between MQLEO compounds and their molecular targets implicated in lung cancer therapy. A compound–target interaction network was constructed using Cytoscape 3.10.2 (Figure 7), enabling visualization of the multi-component regulatory relationships. To prioritize bioactive constituents with potentially significant target interactions, topological analysis was conducted via the cytoNCA plugin, employing the DC algorithm to rank compounds based on their network influence.

The MQLEO compounds were systematically ranked in Table 4 according to their DC values, with ten core constituents exhibiting DC scores surpassing the median threshold (DC ≥ 133). These compounds were identified as candidates of interest for further investigation, reflecting their predicted ability to interact with multiple lung cancer-associated targets.

#### 2.3.4. Pathway Enrichment Analysis of MQLEO’s Core Targets in Lung Cancer

**Figure 8:**
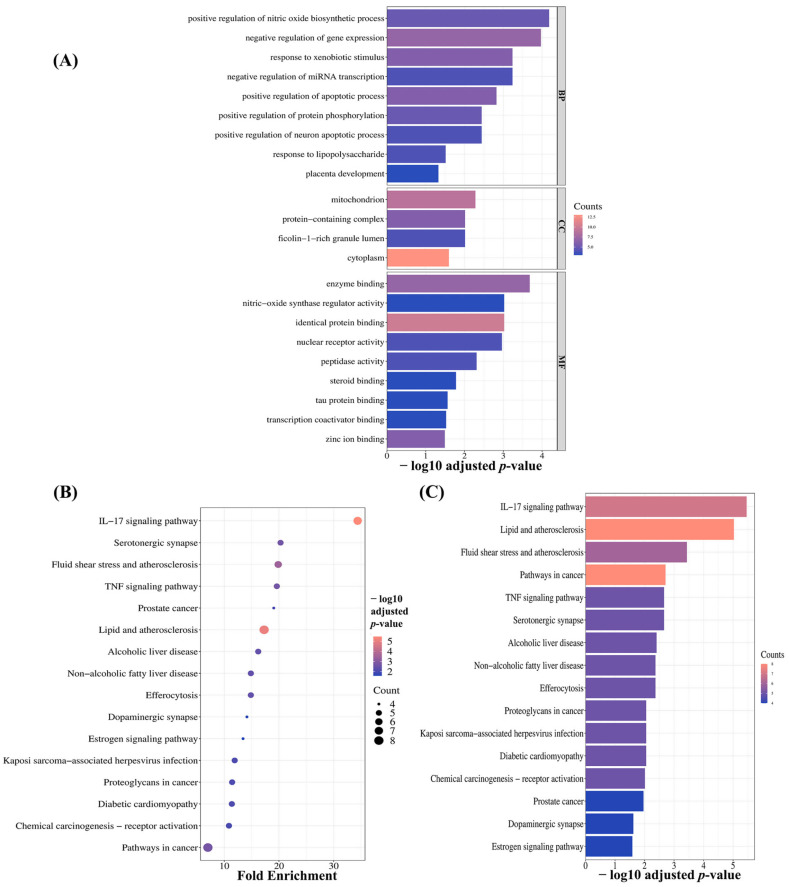
Functional enrichment analysis. (**A**) Significantly enriched Gene Ontology (GO) terms across biological processes (BP), cellular components (CC), and molecular functions (MF). (**B**) Bubble chart and (**C**) bar plot showing significant KEGG pathways.

The therapeutic potential of MQLEO in lung cancer was investigated through pathway enrichment analysis of the 19 identified hub targets. Gene Ontology (GO) enrichment analysis yielded 22 significant terms (adjusted *p* < 0.05) distributed across biological processes (BP; 9 terms), cellular components (CC; 4 terms), and molecular functions (MF; 9 terms), as documented in Supplementary Tables S7–S9 and visualized in Figure 8A.

Among the enriched Bps, several pathways demonstrated particular relevance, including positive regulation of nitric oxide biosynthetic process, negative regulation of gene expression, negative regulation of miRNA transcription, response to xenobiotic stimulus, positive regulation of apoptotic process, and positive regulation of protein phosphorylation. CC analysis revealed significant enrichment in mitochondrial structures, ficolin-1-rich granule lumen, protein-containing complexes, and cytoplasmic regions. The MFs associated with the therapeutic targets encompassed diverse activities, including enzyme binding, nitric-oxide synthase regulator activity, nuclear receptor activity, peptidase activity, transcription coactivator binding, and zinc ion binding.

Further analysis using the Kyoto Encyclopedia of Genes and Genomes (KEGG) framework identified 16 significantly enriched signaling pathways (adjusted *p* < 0.05), as detailed in Supplementary Table S10. Several pathways with established roles in lung cancer pathophysiology emerged from this analysis, including cancer-associated pathways, TNF signaling, IL-17 signaling, chemical carcinogenesis via receptor activation, proteoglycan involvement in cancer, efferocytosis mechanisms, and estrogen signaling (Figure 8B,C).

These comprehensive findings elucidate the complex molecular mechanisms underlying MQLEO’s potential therapeutic efficacy in lung cancer management.

### 2.4. Molecular Docking Evaluation of MQLEO Compounds Against Lung Cancer Targets

**Figure 9:**
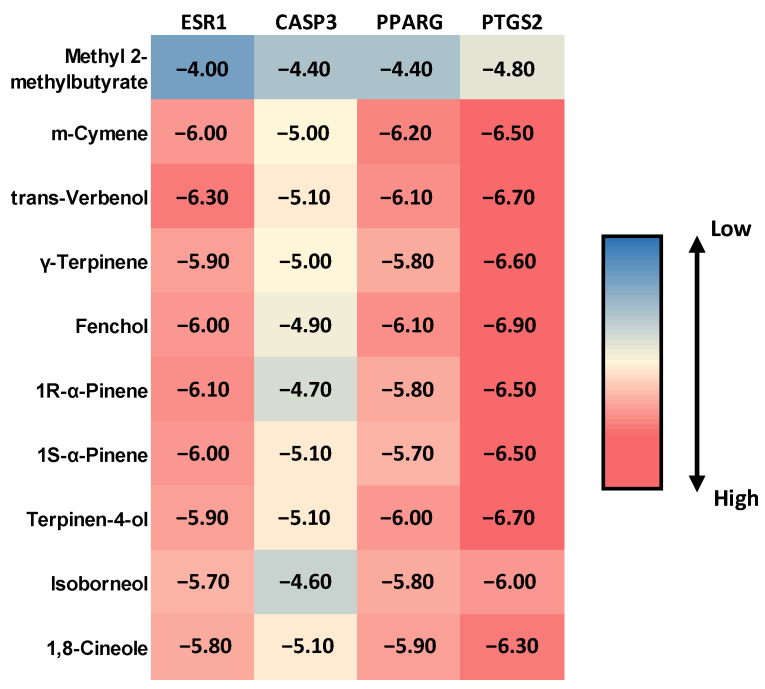
Heatmap illustrating binding energy profiles between *M. quinquenervia* oil compounds (*Y*-axis) and protein targets (*X*-axis) from molecular docking analysis.

Molecular docking analysis was conducted to evaluate the binding affinities of the top 10 MQLEO-derived compounds against four lung cancer-associated targets (ESR1, CASP3, PPARG, and PTGS2). The docking scores (kcal/mol) revealed substantial binding potential across all targets, with values ranging from −4.00 to −6.90 kcal/mol (Figure 9). Notably, PTGS2 exhibited the strongest interactions, with fenchol (−6.90 kcal/mol) and *trans*-verbenol (−6.70 kcal/mol) demonstrating the highest binding affinities. All compounds, except Methyl 2-methylbutyrate, exhibited scores ≤−5.00 kcal/mol for at least one target. This threshold (−5.00 kcal/mol) represents the conventional cutoff for biologically relevant interactions in similar molecular docking studies [36].

Key interactions at the active sites were target-specific (Table 5). For instance, *m*-cymene formed a *pi*-*pi* t-shaped interaction with PHE99 and multiple alkyl bonds (LEU41, LEU44, MET83) in ESR1, while *trans*-verbenol engaged in carbon hydrogen bonds with VAL492 and GLY495 in PTGS2, complemented by hydrophobic interactions with VAL318 and LEU321. *γ*-Terpinene displayed amide–*pi* stacked interactions with ARG80 in PPARG, and terpinen-4-ol formed conventional hydrogen bonds with SER8 in CASP3, suggesting polar complementarity. Structural visualizations (Figures S2–S5) further elucidated distinct binding conformations, reinforcing the computational predictions.

## 3. Discussion

This study re-evaluates the chemical composition of Egyptian-grown *M. quinquenervia* leaf essential oil (MQLEO) using modern GC-MS techniques and assesses its antiproliferative potential through integrated in vitro and computational approaches. Employing network pharmacology and molecular docking transcends the traditional “one-drug/one-target” paradigm to address the complexity of both cancer pathophysiology and MQLEO’s multi-component nature. This systems-level approach is particularly suited for analyzing essential oils containing multiple bioactive compounds that interact with diverse molecular targets simultaneously [45]. Through cytotoxicity screening, migration assays, flow cytometry, and computational analysis, the study uncovers the mechanistic underpinnings of MQLEO’s activity against lung cancer, particularly the A549 cell line. This work advances the understanding of MQLEO’s pharmacological versatility under region-specific conditions and its potential applications in cancer therapeutics.

The GC/MS analysis of *M. quinquenervia* essential oil (MQLEO) derived from Egyptian-grown leaves revealed a distinct phytochemical profile dominated by 1,8-cineole (31.57%), with significant contributions from 1R-*α*-pinene (15.97%), viridiflorol (13.65%), (+)-3-carene (11.57%), and *trans*-ocimenol (8.26%). This composition markedly diverges from the sole prior Egyptian study conducted in 1991, which reported 1,8-cineole as the predominant constituent at 57.2%, alongside α-terpineol (13.2%) and lower α-pinene isomers (6.2% total) [24]. The current findings demonstrate a substantial reduction in 1,8-cineole content (−25.63%), the absence of *α*-terpineol, and a pronounced increase in *α*-pinene isomers (21.26% combined). Notably, sesquiterpene alcohols such as viridiflorol (13.65%) and ledol (4.55%), undetected in earlier analyses, emerged as key constituents, suggesting potential methodological advancements in chromatographic resolution or shifts in plant metabolic pathways over time.

The notable differences in chemical composition between the current study and the 1991 Egyptian analysis may be attributed to the modified extraction protocol. The previous study’s 12 h hydrodistillation versus the current 5 h method likely explains the 25.63% decrease in 1,8-cineole and absence of α-terpineol. Rather than indicating inferior extraction, these differences suggest prevention of the artificial enrichment of 1,8-cineole that occurs during prolonged heating [46,47]. Extended thermal exposure promotes cyclization reactions and the formation of degradation products like α-terpineol [48,49]. The shorter distillation time better preserved the native phytochemical profile, as evidenced by a higher α-pinene isomer content (21.26%) and the detection of previously unreported sesquiterpene alcohols (viridiflorol: 13.65%; ledol: 4.55%). Additionally, advances in GC/MS sensitivity have improved the detection of compounds that may have been below detection thresholds in earlier analyses.

Globally, *M. quinquenervia* exhibits pronounced chemotypic plasticity. Samples from Australia and Papua New Guinea (Chemotype 1) contain high concentrations of E-nerolidol (74–95%) and linalool (14–30%), while Chemotype 2 variants display variable ratios of 1,8-cineole (10–75%), viridiflorol (13–66%), and *β*-caryophyllene (0.5–28%) [22]. Costa Rican samples illustrate organ-specific biosynthesis, with leaf oil aligning partially with our Egyptian profile (1,8-cineole: 31.5%; viridiflorol: 21.7%), while fruit and twig oils emphasize different constituents [23]. East Asian variants show regional variations, with Taiwanese samples exhibiting reduced 1,8-cineole (21.60%) but elevated *α*-pinene (15.93%) [29], whereas Vietnamese samples prioritize 1,8-cineole (42.51%) alongside α-terpineol (12.00%) [12].

These compositional differences arise from a combination of environmental factors (climate, soil type, altitude), genetic influences (chemotype variations), and cultivation practices [50,51]. Such variations critically determine MQLEO’s suitability for applications in aromatherapy, pharmaceuticals, and cosmetics, as therapeutic efficacy is closely linked to specific chemical constituents. A thorough understanding of these variations is therefore essential for selecting essential oils with optimal chemical profiles for targeted applications.

The in vitro evaluation of MQLEO revealed distinct cytotoxic profiles across cancer cell lines. MQLEO demonstrated compliance with NCI criteria for A-549 lung carcinoma cells (IC_50_ = 18.09 μg/mL; selectivity index [SI] = 4.30) [43], fulfilling both cytotoxicity (IC_50_ ≤ 30 μg/mL) and selectivity (SI > 3) requirements. This dual advantage of the highest potency and selectivity provided the rationale for selecting A-549 cells for subsequent antiproliferative assays, underscoring MQLEO’s therapeutic potential specifically for lung cancer. While MQLEO also exhibited cytotoxicity against MCF-7 breast cancer cells (IC_50_ = 27.74 μg/mL), its reduced selectivity (SI = 2.80) suggests limited specificity for this lineage. The notably low SI for HepG-2 hepatocellular carcinoma (1.18) further emphasizes cell line-dependent efficacy.

It is important to acknowledge that Staurosporine, used as a positive control, demonstrated higher cytotoxic activity across all cell lines (IC_50_ values: 3.92–4.62 μg/mL), as expected from this potent protein kinase inhibitor [52]. This comparison primarily validates the experimental methodology rather than serving as a therapeutic benchmark. Notably, MQLEO showed lower cytotoxicity toward non-cancerous VERO cells (CC_50_ = 77.76 μg/mL vs. Staurosporine’s 24.20 μg/mL), highlighting its favorable safety profile. MQLEO’s activity represents promising potential for a natural product complex mixture, particularly considering its enhanced selectivity toward cancer cells and multi-target mechanistic profile.

Contextualizing the cytotoxic activity of MQLEO against A-549 lung carcinoma cells (IC_50_ = 18.09 μg/mL) within essential oil research, its potency aligns well with other terpenoid-rich essential oils from the Myrtaceae family known for their anticancer properties. For instance, the essential oil of *Myrcia splendens* demonstrated significant cytotoxicity against A549 cells, reducing colony formation and migration, with an IC_50_ value of 20.14 μg/mL, highlighting similar antiproliferative potential [53]. Likewise, essential oils from *M. viminalis* and *M. armillaris* showed cytotoxic effects on A549 cells with IC_50_ values of 24.12 μg/mL and 10.2 μg/mL, respectively [16], further supporting the therapeutic relevance of Myrtaceae-derived oils in lung cancer. These comparisons indicate that MQLEO’s activity is consistent with the cytotoxic profiles of other Myrtaceae essential oils, underscoring its promising role while situating it within a broader context of similar natural products with anticancer potential.

MQLEO significantly impeded A549 cell migration, as evidenced by the time-dependent suppression of wound closure (62% at 24 h; 68% at 72 h). This sustained anti-migratory effect implies interference with cytoskeletal dynamics, adhesion signaling, or metastasis-associated pathways, such as matrix metalloproteinase (MMP) regulation or integrin-mediated processes [54,55]. The inhibition of cellular regeneration and matrix formation further suggests a capacity to attenuate invasiveness, a hallmark of aggressive malignancies. The durability of this suppression contrasts with the transient effects observed for other agents, positioning MQLEO as a promising anti-metastatic candidate [56,57].

MQLEO induced marked apoptosis in A549 cells, elevating the total cell death 10.6-fold relative to controls, predominantly through apoptotic mechanisms (24.32% vs. 0.7% in controls). The marginal necrosis increase (3.29% vs. 1.91%) suggests partial contribution from non-apoptotic pathways, potentially linked to membrane disruption at higher concentrations [58]. Concurrent G_0_-G_1_ phase arrest implicates the modulation of cyclin-dependent kinase (CDK) activity or the upregulation of cyclin inhibitors (e.g., p21), mechanisms distinct from DNA-damaging or microtubule-targeting agents [59,60].

While apoptosis is favored for its controlled nature, the observed necrosis warrants mechanistic differentiation to optimize therapeutic specificity. These findings collectively underscore MQLEO’s multifactorial anticancer potential, with a particular focus on lung cancer applications.

The Annexin V/PI flow cytometry data provide quantitative evidence of apoptosis induction by MQLEO in A549 cells. Direct visualization of nuclear morphological changes through fluorescence microscopy with chromatin dyes would offer complementary verification of the apoptotic process. Therefore, future studies should incorporate time-course fluorescent microscopy with nuclear stains (e.g., Hoechst 33342 or DAPI) to confirm characteristic apoptotic features such as chromatin condensation and nuclear fragmentation. This multi-method approach would strengthen the mechanistic understanding of MQLEO’s pro-apoptotic effects and provide temporal resolution of the cell death process.

The integration of network pharmacology and computational biology provides a systematic framework to elucidate MQLEO’s multi-target therapeutic potential. Our findings underscore its poly-pharmacological nature, characterized by bioactive constituents’ adherence to drug-like properties and synergistic interactions with lung cancer-associated targets.

All 19 MQLEO-derived compounds comply with Lipinski’s rule of five, demonstrating favorable pharmacokinetic properties such as gastrointestinal absorption and oral bioavailability. The identification of 317 molecular targets shared between MQLEO compounds and lung cancer pathogenesis highlights a robust mechanistic overlap, suggesting the modulation of critical pathways including apoptosis, proliferation, and inflammation [61].

The PPI network analysis identified ESR1, CASP3, PPARG, and PTGS2 as top-ranking hub genes. ESR1 (estrogen receptor alpha), a nuclear hormone receptor, has emerged as a critical mediator in lung cancer progression, particularly in non-small cell lung cancer (NSCLC). While lung cancer is not traditionally classified as hormone-dependent, ESR1 signaling has been implicated in tumor proliferation, survival, and metastasis through estrogen-mediated pathways, with overexpression correlating with poor prognosis in certain subtypes [62]. The inclusion of ESR1 as a hub gene suggests that MQLEO may interfere with estrogen receptor signaling, potentially attenuating oncogenic pathways such as MAPK/ERK or PI3K/AKT, which are frequently dysregulated in lung cancer [63,64]. CASP3, a key executor of apoptosis frequently suppressed in cancer cells [65,66], suggests that MQLEO may restore apoptotic signaling [67,68]. Similarly, PPARG, a regulator of cell differentiation and metabolism, exhibits tumor-suppressive roles in NSCLC models [69,70], while PTGS2 (COX-2) drives inflammation-linked carcinogenesis [71,72,73]

Topological analysis identified ten MQLEO constituents with high-degree centrality (DC ≥ 133), including Methyl 2-methylbutyrate; *m*-cymene; *trans*-verbenol; *γ*-terpinene; fenchol; 1R-*α*-pinene; 1S-*α*-pinene; terpinen-4-ol; isoborneol; and 1,8-cineole. These terpenoid compounds are well-documented for their anti-inflammatory, pro-apoptotic, and antioxidant properties in cancer models [74,75,76,77,78,79,80,81].

The GO and KEGG analyses revealed a multifaceted mode of action, implicating diverse biological processes, cellular components, and signaling pathways that align with established hallmarks of lung cancer pathogenesis [82,83].

The enrichment of biological processes such as the positive regulation of the nitric oxide biosynthetic process and apoptotic process underscores MQLEO’s potential to modulate key pathways involved in tumor suppression. Nitric oxide (NO) plays a dual role in cancer biology, acting as both a pro- and anti-tumor agent depending on concentration and context. Its upregulation here suggests that MQLEO may promote anti-tumor effects by inducing oxidative stress or enhancing immune-mediated cytotoxicity in malignant cells [84,85]. Furthermore, the enrichment of the positive regulation of apoptosis aligns with the therapeutic goal of triggering programmed cell death in cancer cells, a mechanism exploited by many chemotherapeutic agents [5,86]. The concurrent activation of protein phosphorylation pathways highlights MQLEO’s potential to regulate signaling cascades, such as those mediated by kinases, which are frequently dysregulated in lung cancer progression and metastasis [87,88].

The significant enrichment of cellular components in mitochondrial structures and cytoplasmic regions further supports the involvement of mitochondrial-mediated apoptosis, a critical pathway disrupted in many cancers [89]. Mitochondria are central to energy metabolism and apoptotic signaling, and their dysregulation is a hallmark of cancer cell survival. MQLEO’s interaction with these compartments may disrupt cancer cell homeostasis, promoting cell death [90,91,92]. Additionally, the association with protein-containing complexes suggests a role in modulating macromolecular assemblies, potentially interfering with oncogenic signaling networks or stabilizing tumor-suppressive complexes [93].

Molecular functions such as enzyme binding, nitric-oxide synthase regulator activity, and zinc ion binding further elucidate MQLEO’s mechanistic versatility. The ability to bind enzymes and regulate nitric oxide synthesis may enhance its capacity to interfere with metabolic reprogramming in cancer cells [94,95]. Zinc ion binding, essential for the structural and functional integrity of numerous proteins, could stabilize transcription factors or enzymes critical for maintaining genomic stability, thereby counteracting oncogenic drivers [96,97,98].

KEGG pathway analysis identified signaling cascades including TNF, IL-17, and estrogen signaling. The TNF and IL-17 pathways mediate inflammation-driven tumorigenesis [99,100,101], suggesting that MQLEO may attenuate the pro-tumorigenic microenvironment. Pathways linked to chemical carcinogenesis and proteoglycans in cancer highlight a potential disruption of extracellular matrix remodeling and receptor tyrosine kinase signaling, key drivers of tumor invasion [102,103,104,105,106]. Notably, the involvement of efferocytosis mechanisms, a process by which apoptotic cells are cleared [107], suggests MQLEO may enhance immune surveillance by promoting the recognition and elimination of malignant cells, thereby limiting tumor immune escape [108].

The molecular docking results underscore the potential of MQLEO-derived compounds as multi-target agents, particularly through the modulation of inflammatory and apoptotic pathways. PTGS2 emerged as a critical target, with compounds such as fenchol, *trans*-verbenol, and terpinen-4-ol demonstrating high binding affinities (docking scores of −6.90, −6.70, and −6.70 kcal/mol, respectively). These interactions likely disrupt prostaglandin-mediated inflammatory processes that contribute to tumorigenesis [109].

*γ*-Terpinene exhibited a robust binding profile with PTGS2 (docking score of −6.60 kcal/mol) and moderate affinities with ESR1 and PPARG. Its interactions, primarily mediated through hydrophobic and *pi*-alkyl contacts, support its potential to modulate multiple signaling pathways.

Several compounds, including *m*-cymene and *trans*-verbenol, demonstrated polypharmacological characteristics by interacting with multiple targets Via a combination of hydrophobic contacts, *pi*-*pi* stacking, and hydrogen bonding. Such simultaneous modulation of diverse cellular pathways could be particularly advantageous in lung cancer treatment.

Stereochemical variations were evident between 1R-*α*-pinene and 1S-*α*-pinene, with 1S-*α*-pinene showing unique binding characteristics with CASP3. This underscores the significance of molecular conformation in ligand–protein recognition and the potential for stereochemistry-driven optimization in future drug design.

Despite being a major constituent of MQLEO, 1,8-cineole exhibited only modest binding effects across evaluated targets (docking scores from −5.80 to −6.30 kcal/mol), suggesting it may function through chemopreventive or adjuvant mechanisms rather than as a primary inhibitor. Conversely, methyl 2-methylbutyrate, a compound prioritized by network pharmacology, exhibited weaker docking scores (−4.00 to −4.80 kcal/mol), suggesting that its therapeutic contribution may involve indirect mechanisms such as allosteric modulation or synergistic interactions with other MQLEO compounds. Its structural attributes, including a short aliphatic chain and an ester functional group, might limit direct binding to the target sites while enhancing systemic bioavailability or interactions with broader signaling pathways.

In summary, this comprehensive in silico analysis identifies key compounds in MQLEO that collectively contribute to its significant anticancer activity against A549 lung adenocarcinoma cells through poly-pharmacological mechanisms. This complex mixture contains bioactive monoterpenes (1,8-cineole [31.57%], *α*-pinene isomers, *γ*-terpinene, terpinen-4-ol, *trans*-verbenol, fenchol, m-cymene, and isoborneol), and sesquiterpenes (viridiflorol and ledol) that simultaneously modulate multiple oncogenic pathways. Unlike conventional chemotherapeutics targeting single molecular entities with high potency, MQLEO’s therapeutic potential derives from cumulative and likely synergistic effects. Notably, while individual compounds like 1,8-cineole show only moderate binding affinity in docking studies and methyl 2-methylbutyrate exhibits high network centrality despite weak direct binding, the complete oil demonstrates significant cytotoxicity, suggesting the importance of compound interactions rather than individual effects.

Previous studies have reported these compounds target cancer through complementary mechanisms. 1,8-cineole suppresses NF-κB-mediated inflammatory signaling and induces apoptosis [74,80,81]. Terpinen-4-ol modulates oxidative stress and mitochondrial function [79]. *γ*-Terpinene and *α*-pinene isomers provide chemopreventive activity via ROS scavenging and caspase-3 activation [77,78]. *Trans*-verbenol displays selective cytotoxicity against tumor cells with minimal toxicity to non-neoplastic cells [76], through mechanisms including cell membrane destabilization and mitochondrial dysfunction [75].

By simultaneously targeting multiple cancer hallmarks and potentially enhancing each other’s bioavailability and cellular uptake, these compounds create a comprehensive anticancer effect that may offer advantages in overcoming the treatment resistance commonly encountered with single-target therapies. To definitively establish the nature of these interactions, subsequent investigations should employ methodologies such as the combination index and differential gene expression profiling to quantitatively compare the effects of whole MQLEO against those of its isolated constituents.

Future research should focus on isolating and validating MQLEO’s bioactive components in experimental lung cancer models, elucidating molecular pathways involving Bcl-2 proteins, caspases, and cell cycle regulators. Parallel studies should examine potential synergies with conventional treatments. Advancing clinical translation requires addressing pharmacokinetics, bioavailability, and tumor microenvironment interactions through comprehensive preclinical models. Compound optimization strategies should incorporate dose–response profiling, stereochemical refinement, and rigorous toxicological evaluation. These multidisciplinary efforts are critical to transitioning MQLEO-derived agents from promising in silico and in vitro data into clinically viable therapies targeting inflammatory and apoptotic pathways in lung cancer.

## 4. Materials and Methods

### 4.1. Botanical Specimen Collection and Processing Methodology

**Figure 10:**
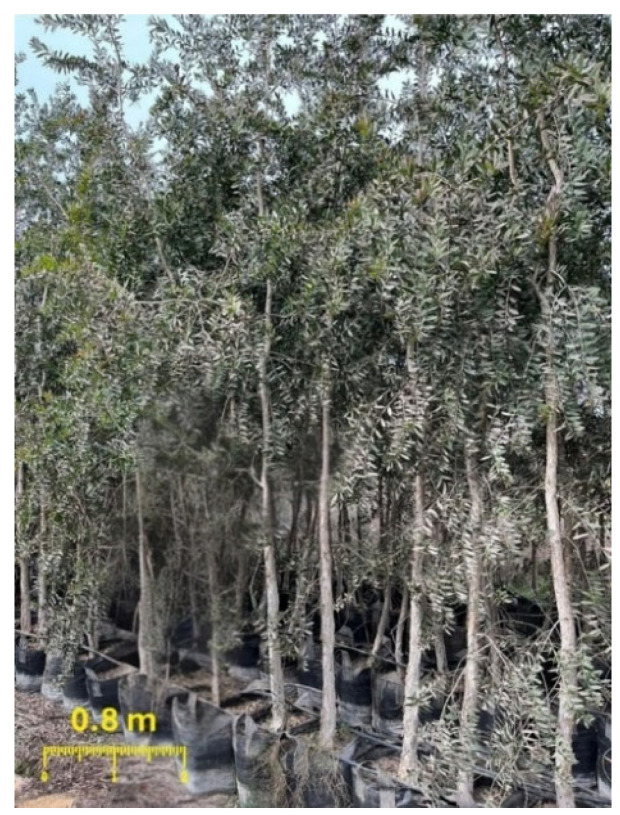
*Melaleuca quinquenervia* (Cav.) S.T. Blake.

In January 2025, fresh leaves of *Melaleuca quinquenervia* (Cav.) S.T. Blake (Figure 10) were obtained from the nursery of Agricultural Engineer Ahmed Mohamed Abdel Aaty, located in Shanbari, Oseem, Giza, Egypt. The plant species was authenticated by Eng. Therese Labib, a recognized taxonomist affiliated with the Ministry of Agriculture and formerly the head of El-Orman Botanical Garden. A representative voucher specimen (ZU-Ph-Cog-0521) was cataloged in the Herbarium of the Pharmacognosy Department at Zagazig University’s Faculty of Pharmacy for archival purposes.

Approximately 600 g of fresh leaves were subjected to conventional hydrodistillation via a Clevenger apparatus operating at ambient pressure. The extraction process, conducted at ~100 °C for 5 h, yielded essential oil, which was subsequently dehydrated using anhydrous sodium sulfate to eliminate moisture. The purified oil was then transferred to light-protected glass vials and refrigerated at 4 °C to maintain stability prior to downstream chemical and bioactivity assessments.

**Figure 11:**
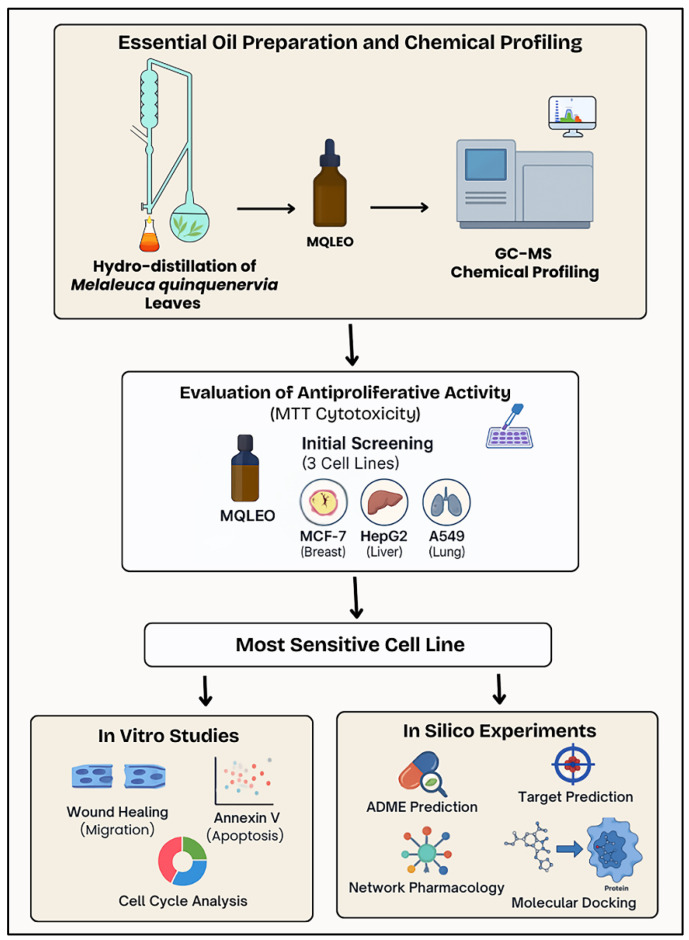
Schematic representation of the experimental workflow employed in the investigation of *Melaleuca quinquenervia* leaf essential oil.

A comprehensive schematic representation of the experimental workflow, from essential oil extraction and chemical profiling to biological screening and in silico analyses, is illustrated in Figure 11.

### 4.2. GC–MS Characterization of MQLEO

Chromatographic profiling of MQLEO was performed using a Shimadzu GCMS-QP2010 system (Kyoto, Japan) equipped with an Rtx-5MS fused silica capillary column (30 m × 0.25 mm ID, 0.25 μm film thickness; Restek, Bellefonte, PA, USA) and a split/splitless injector. The temperature protocol was initiated with a 2 min isothermal hold at 45 °C, followed by a linear temperature ramp of 5 °C/min to 300 °C, and a final 5 min hold at 300 °C. Helium (1.41 mL/min flow rate) served as the carrier gas, with the injector temperature fixed at 250 °C.

Mass spectrometric parameters included a scan range of 35–500 *m*/*z*, ionization voltage of 70 eV, filament emission current of 60 mA, and ion source temperature of 200 °C. Samples were diluted to 1% (*v*/*v*) in hexane, and 1 μL aliquots were injected at a 1:15 split ratio. Compound identification was accomplished by cross-referencing experimental retention indices (RIs) and mass spectra against authenticated standards and databases, including the Adams Library [33], NIST11/2011/EPA/NIH, Wiley 10th Edition, and published literature [17,34,35,36,37,38,39,40,41,42]. RIs were derived using a homologous series of n-alkanes (C_8_–C_28_) analyzed under identical conditions. Quantified constituents and their relative abundances are summarized in Table 1.

### 4.3. In Vitro Validations

#### 4.3.1. Cytotoxicity Screening

The cytotoxic effects of MQLEO were investigated against three human carcinoma cell lines, HepG2 (liver), MCF-7 (breast), and A549 (lung), as well as Vero cells as a non-cancerous control, using the standard MTT assay protocol [110].

##### Cell Culture and Treatment

Cell lines, acquired from the American Type Culture Collection (ATCC), were maintained in DMEM (Invitrogen/Life Technologies, Carlsbad, CA, USA) supplemented with 10% fetal bovine serum (FBS; HyClone, Cytiva, Logan, UT, USA), 10 μg/mL insulin, and 1% penicillin–streptomycin. For experiments, cells (10^3^ cells/well) were seeded in 96-well plates with 100 μL complete growth medium and incubated overnight at 37 °C. Subsequently, cells were treated with MQLEO using a 2-fold (1:2) serial dilution series starting from a 100 μg/mL stock to achieve final concentrations of 100, 50, 25, 12.5, 6.25, 3.13, 1.56, 0.78, and 0.39 μg/mL dissolved in 0.5% dimethyl sulfoxide (DMSO) and incubated for 48 h under identical conditions. Wells containing 0.5% DMSO served as the negative control. Staurosporine was used as a positive control to validate the assays, confirming the reliability of the methods.

##### MTT Assay Protocol

Following treatment, 20 μL of MTT reagent (5 mg/mL) was added to each well, and plates were incubated for 6 h at 37 °C. The formazan crystals formed were dissolved using DMSO, and absorbance was measured at 570 nm using a microplate reader (SunRise, TECAN Inc., Morrisville, NC, USA). Cell viability was calculated as follows:

Viability (%) = (OD_c_/OD_t_) × 100% (1)

where OD_t_ and OD_c_ represent the mean optical densities of treated and untreated cells, respectively.

##### Data Analysis and Statistical Evaluation

Dose–response curves (Figure S6) were generated by plotting cell viability against MQLEO concentrations. The half-maximal inhibitory concentration (IC_50_) for cancer cells and the 50% cytotoxic concentration (CC_50_) for Vero cells were determined using nonlinear regression analysis in GraphPad Prism version 9.5.1.733 (San Diego, CA, USA) [111]. Data from triplicate experiments are expressed as mean ± standard deviation (SD).

From these analyses, the IC_25_ value for MQLEO against A549 cells was determined to be 0.70 μg/mL.

##### Selectivity Index (SI)

The therapeutic selectivity of MQLEO was assessed by calculating the SI for each cancer cell line:

SI = IC_50_(Cancer Cell Line)/CC_50_(VERO) (2)

#### 4.3.2. Evaluation of Anti-Migratory Effects of MQLEO on A549 Tumor Cells

The inhibitory effect of MQLEO on the migratory capacity of A549 lung carcinoma cells was assessed using a wound healing assay. Briefly, cells were seeded into 12-well microtiter plates at a density of 4 × 10^6^ cells per well and cultured until a confluent monolayer formed. A standardized wound was introduced into the monolayer using a sterile pipette tip, followed by gentle rinsing with phosphate-buffered saline (PBS) to remove dislodged cells. Fresh culture medium containing MQLEO at the IC_25_ concentration (0.70 μg/mL) was added to the treatment groups, while control groups received medium supplemented with 0.5% DMSO. All plates were incubated at 37 °C under 5% CO_2_. Wound closure dynamics were monitored at regular intervals using phase-contrast microscopy. The quantitative analysis of migration inhibition was performed by measuring the residual scratch area in treated cells relative to untreated controls, with healing efficiency expressed as the percentage reduction in wound area over time.

#### 4.3.3. Apoptosis and Cell Cycle Analysis of A549 Cells Treated with MQLEO

The pro-apoptotic effects of MQLEO on A549 lung carcinoma cells were evaluated using an Annexin V-FITC/PI Apoptosis Detection Kit (BioVision, Mountain View, CA, USA, Catalog #K101-25). This assay quantifies early apoptotic events by detecting phosphatidylserine (PS) externalization, a hallmark of apoptosis initiation, through Annexin V binding. Briefly, cells were seeded in 96-well plates at a density of 2 × 10⁷ cells/well, exposed to MQLEO at the IC_25_ concentration (0.70 μg/mL) for 24 h, and subsequently harvested. Cells were washed with phosphate-buffered saline (PBS) and resuspended in 200 μL of 1X Annexin-binding buffer. Annexin V-FITC (5 μL) and propidium iodide (PI, 10 μL) were added to the suspension, followed by a 15 min incubation in the dark. Fluorescence signals were acquired via flow cytometry (excitation: λ = 488 nm; emission: λ = 530 nm for FITC), distinguishing viable (Annexin V^−^/PI^−^), early apoptotic (Annexin V^+^/PI^−^), late apoptotic (Annexin V^+^/PI^+^), and necrotic (Annexin V^−^/PI^+^) populations.

For cell cycle distribution analysis, a Propidium Iodide (PI) Staining Kit (Abcam, Cambridge, UK, Catalog #ab139418) was employed. A549 cells were seeded in 48-well plates, allowed to adhere for 12 h at 37 °C under 5% CO_2_, and treated with MQLEO at the IC_25_ concentration (0.70 μg/mL) for 48 h. Cells were pelleted by centrifugation (2000 rpm, 5 min), fixed in ice-cold 70% ethanol (2 h, 4 °C), and rehydrated in PBS. Prior to analysis, cells were incubated in PI staining solution (5 μg/mL PI, 10 μg/mL RNase A) for 30 min in the dark to label nuclear DNA. Flow cytometry was performed (excitation: λ = 493 nm; emission: λ = 636 nm) and cell cycle phase percentages (G0/G1, S, G2/M) were quantified using FlowJo™ software, version 10.8.1 (FlowJo LLC, Ashland, OR, USA; distributed by BD Biosciences, Gurgaon, Haryana, India). Data were normalized to untreated controls to assess MQLEO-induced disruptions in cell cycle progression.

##### Statistical Analysis

All quantitative data are presented as the mean ± standard deviation (SD) of three independent experiments (*n* = 3). Statistical comparisons between two independent groups (MQLEO-treated vs. A549 control at each time point) were performed using an unpaired, two-tailed Student’s t-test with Welch’s correction to account for unequal variances. A *p*-value < 0.05 was considered statistically significant. All analyses and graphical presentations were carried out in GraphPad Prism version 9.5.1.733 (San Diego, CA, USA).

### 4.4. Network Pharmacology

#### 4.4.1. Pharmacokinetic Evaluation of MQLEO Phytoconstituents

The canonical SMILES representations of the 19 phytoconstituents, identified Via GC-MS analysis, were obtained from the PubChem database (https://pubchem.ncbi.nlm.nih.gov/, accessed on 25 January 2025) or generated using ChemDraw v22.0.0.22 (PerkinElmer Informatics, Inc., Buckinghamshire, UK). These SMILES notations were subsequently analyzed using the SwissADME web server to assess drug-like properties. The evaluation employed specific screening criteria, including Lipinski’s rule of five [112] and a threshold Abbott oral bioavailability score exceeding 0.5, to determine their pharmaceutical potential.

#### 4.4.2. Identification of Intersection Genes Between Lung Cancer and MQLEO Bioactive Compounds

Putative targets associated with the compounds were predicted through the Swiss Target Prediction (STP) (http://www.swisstargetprediction.ch/, accessed on 27 January 2025) [113] and TargetNet databases (http://targetnet.scbdd.com/, accessed on 28 January 2025) [114]. Additionally, targets linked to lung cancer were gathered from three sources: Online Mendelian Inheritance in Man (OMIM, https://www.omim.org/, accessed on 30 January 2025) [115], DisGeNet (https://www.disgenet.org/search, accessed on 30 January 2025), and GeneCards (https://www.genecards.org/, accessed on 30 January 2025) [116,117], using the keyword “Lung Cancer.” UniProt (https://www.uniprot.org/, accessed on 31 January 2025) [118] was then employed to obtain the corresponding UniProt IDs and gene symbols for these targets. To ensure data accuracy, duplicate entries were removed. Finally, Venny 2.1.0 (https://bioinfogp.cnb.csic.es/tools/venny/, accessed on 31 January 2025) was used to identify and visualize the overlapping target genes associated with both the bioactive compounds and lung cancer.

#### 4.4.3. PPI Network Construction

After identifying shared targets, protein–protein interactions were analyzed using the STRING database (v12.0; https://string-db.org/; accessed 2 February 2025) [119], with a confidence threshold > 0.4 and the search restricted to Homo sapiens. The interaction dataset was imported into Cytoscape software, version 3.10.2 (Cytoscape Consortium; Institute for Systems Biology, Seattle, WA, USA [120] for network visualization and topology analysis. Network centrality metrics—including betweenness centrality (BC, indicating a node’s bridging role), closeness centrality (CC, reflecting proximity to other nodes), and degree centrality (DC, quantifying direct connections per node)—were calculated using the CytoNCA plugin [36,121].

Hub targets were identified through a two-tiered screening approach: First, nodes with DC values ≥ 2× the median were retained. Second, core targets were filtered by retaining nodes exceeding or equaling median values for BC, CC, and DC [36]. This strategy prioritized highly interconnected nodes with pivotal roles in network integrity, potentially representing critical regulators in disease pathogenesis or therapeutic candidates.

#### 4.4.4. Compound–Target Interaction Network Construction

To illustrate compound–target relationships, an interaction network was constructed using Cytoscape software (v3.10.2) to map associations between MQLEO bioactive constituents and their putative therapeutic targets in lung cancer. In this network, compounds and targets were represented as nodes, while edges denoted their interactions. The CytoHubba plugin [122] was utilized to evaluate node centrality, with degree centrality serving as the primary metric to prioritize compounds exhibiting the highest connectivity. Key bioactive constituents were identified by filtering nodes with degree values exceeding the median threshold, emphasizing their potential therapeutic relevance in modulating lung cancer-related pathways.

#### 4.4.5. Functional Annotation and Pathway Enrichment

Core therapeutic targets were functionally characterized through GO and KEGG pathway enrichment analyses. Hub genes were submitted to the DAVID bioinformatics platform (https://david.ncifcrf.gov/; accessed 5 February 2025) [123] to annotate molecular functions (MF), biological processes (BP), and cellular components (CC), as well as to identify enriched KEGG pathways. Analyses were restricted to Homo sapiens, significant terms were determined Via Benjamini–Hochberg correction (adjusted *p* < 0.05), and results were graphically represented through bar plots and bubble charts generated by the Bioinformatics Online Platform (http://www.bioinformatics.com.cn/en; accessed 7 February 2025) to facilitate data interpretation and highlight pathway relevance.

### 4.5. Molecular Docking Analysis

The top four core target proteins were selected for molecular docking studies with the ten key bioactive constituents of MQLEO, with binding energies calculated to assess interaction strength. These protein targets (ESR1, CASP3, PPARG, and PTGS2) were specifically chosen based on their highest degree centrality (DC) values (ESR1: DC = 99; CASP3: DC = 93; PPARG: DC = 88; PTGS2: DC = 85) from our PPI network analysis (Table 3), identifying them as the most critical nodes mediating MQLEO’s potential therapeutic effects after the two-tier filtration process of 315 overlapping targets. The three-dimensional crystal structures of Estrogen receptor (ESR1; PDB ID: 2OUZ; resolution: 2.00 Å) [124], caspase-3 (CASP3; PDB ID: 5I9B; 1.80 Å) [125], peroxisome proliferator-activated receptor gamma (PPARG; PDB ID: 8ATY; 1.90 Å) [126], and prostaglandin G/H synthase 2 (PTGS2; PDB ID: 5F19; 2.04 Å) [127] were retrieved from the Protein Data Bank (https://www.rcsb.org; accessed 10 February 2025) [128]. Protein structures were preprocessed using UCSF Chimera (v1.17.3) [129], following established protocols [130].

Binding pockets for molecular docking were determined through a computational approach. Ligand-binding pockets were predicted via the CASTp server (http://sts.bioe.uic.edu/castp/; accessed 11 February 2025) [131], with grid coordinates derived from identified active sites. For ESR1, PPARG, and PTGS2, the binding sites were further validated by the presence of co-crystallized ligands in their respective PDB structures. For CASP3, where no co-crystallized ligand was available, pocket selection relied solely on CASTp’s quantitative pocket analysis to identify the most favorable binding region. This data-driven approach ensured that the selected binding pockets were structurally appropriate for each target protein. The grid specifications (center coordinates, dimensions, and active site residues) are detailed in Supplementary Table S11.

The 3D structures of MQLEO compounds, sourced from PubChem, were converted into pdbqt format using OpenBabel (v2.4.1) [132]. AutoDock Vina (v1.1.2), integrated into UCSF Chimera, was employed to define grid boxes around each protein’s active site and perform molecular docking under default parameters, generating 10 ligand conformations per compound. Grid specifications (center coordinates, dimensions, and active site residues) are detailed in Supplementary Table S11.

Docking simulations produced ten binding poses per ligand, with scoring based on binding free energy (kcal/mol). The conformation with the lowest binding energy and acceptable root mean square deviation (RMSD) was considered optimal. Protein–ligand interactions were analyzed using Biovia Discovery Studio Visualizer (v21.1.0.20298) [133], focusing on hydrogen bonding, hydrophobic contacts, and steric complementarity to elucidate binding mechanisms.

## 5. Conclusions

*M. quinquenervia* leaf essential oil (MQLEO) exhibited a unique phytochemical profile distinct from previous reports and demonstrated significant bioactivity against A549 lung cancer cells. MQLEO showed selective cytotoxicity toward lung cancer cells while sparing normal cells, and effectively inhibited cancer cell migration, induced apoptosis, and triggered G_0_/G_1_ phase cell cycle arrest. Network pharmacology and molecular docking analyses revealed that MQLEO compounds interact with multiple targets (ESR1, CASP3, PPARG, PTGS2), suggesting mechanisms involving the modulation of inflammation, apoptosis, and estrogen signaling. These findings underscore MQLEO’s potential as a multi-target natural product with anticancer properties within a systems pharmacology framework. Nevertheless, in vivo validation and pharmacokinetic studies are required to confirm its efficacy, safety, and bioavailability for clinical applications.

## Figures and Tables

**Table 1:** Chemical composition of *M. quinquenervia* leaf essential oil.

Peak	Rt	Compound Name	Chemical Class	RI_Exp._ ^a^	RI_Lit._ ^b^	Area %	Identification ^c^
1.	3.540	Methyl 2-methylbutyrate (Butanoic acid, 2-methyl-, methyl ester)	Fatty acid ester	769	767	0.04	MS, RI
2.	7.565	1R-*α*-Pinene (D-α-Pinene)	Bicyclic monoterpene hydrocarbon	923	922	15.97	MS, RI
3.	8.725	1S-*α*-Pinene (L-α-Pinene)	Bicyclic monoterpene hydrocarbon	962	963	5.29	MS, RI
4.	10.075	*m*-Cymene	Aromatic monoterpene hydrocarbon	1006	1005	1.54	MS, RI
5.	10.305	1,8-Cineole (Eucalyptol)	Monocyclic monoterpene oxide	1013	1013	31.57	MS, RI
6.	10.370	(+)-3-Carene	Aromatic monoterpene hydrocarbon	1015	1015	11.57	MS, RI
7.	11.230	γ-Terpinene	Monocyclic monoterpene hydrocarbon	1043	1042	0.58	MS, RI
8.	12.750	Fenchol	Monocyclic monoterpene alcohol	1091	1097	0.24	MS, RI
9.	14.275	Isoborneol	Bicyclic monoterpene alcohol	1141	1147	0.48	MS, RI
10.	14.680	Terpinen-4-ol	Monocyclic monoterpene alcohol	1154	1152	0.80	MS, RI
11.	14.950	*trans*-Verbenol	Bicyclic monoterpene alcohol	1163	1155	0.11	MS, RI
12.	15.065	*trans*-Ocimenol	Acyclic monoterpene alcohol	1167	1169	8.26	MS, RI
13.	22.955	*α*-Guaiene	Bicyclic sesquiterpene hydrocarbon	1445	1440	0.16	MS, RI
14.	23.860	(+)-Ledene	Bicyclic sesquiterpene hydrocarbon	1480	1482	0.24	MS, RI
15.	25.590	(−)-Palustrol	Bicyclic sesquiterpene alcohol	1561	1562	0.56	MS, RI
16.	25.795	Caryophyllene oxide	Tricyclic Sesquiterpene oxide	1572	1578	2.23	MS, RI
17.	25.930	(−)-Globulol	Tricyclic sesquiterpene alcohol	1578	1580	0.25	MS, RI
18.	26.135	Viridiflorol	Tricyclic sesquiterpene alcohol	1589	1587	13.65	MS, RI
19.	26.390	Ledol	Tricyclic sesquiterpene alcohol	1601	1599	4.55	MS, RI
		Total identified				98.05	
		Monoterpenes hydrocarbons				34.95	
		Oxygenated monoterpenes				41.46	
		Sesquiterpene hydrocarbons				0.40	
		Oxygenated sesquiterpenes				21.24	

^a^ Experimentally determined retention indices (RI) on the Rtx-5MS column, calibrated against C_8_–C_28_ n-alkane standards. ^b^ Reference RI values sourced from established literature. ^c^ Compound identification was verified through a comparative analysis of mass spectrometry (MS) data and RI values using authenticated reference databases, including the Adams library [33], NIST 11, Wiley Registry 10th edition, and corroborated by published scientific literature [17,34,35,36,37,38,39,40,41,42].

**Table 2:** Cytotoxic effects of *M. quinquenervia* leaf essential oil and Staurosporine on VERO, MCF-7, HepG-2, and A-549. Values shown as CC_50_, IC_50_ (Mean ± SD), and selectivity index (SI).

	Vero	MCF-7		HepG-2		A-549	
	CC_50_	IC_50_	SI	IC_50_	SI	IC_50_	SI
MQLEO	77.76 ± 3.96	27.74 ± 1.41	2.80	66.04 ± 3.36	1.18	18.09 ± 0.92	4.30
Staurosporine	24.20 ± 1.23	4.62 ± 0.24	5.24	9.51 ± 0.48	2.54	3.92 ± 0.2	6.17

**Table 3:** Core protein targets identified through PPI network analysis and their topological centrality metrics (BC: betweenness centrality; CC: closeness centrality; DC: degree centrality).

Target Name	BC	CC	DC
ESR1	8287.888526	0.26860565	99
CASP3	4448.232593	0.2670068	93
PPARG	5617.635482	0.2652027	88
PTGS2	5493.159146	0.26430976	85
HSP90AA1	3015.111312	0.26079734	82
MMP9	3298.324704	0.25950413	82
GSK3B	3013.523609	0.26036484	71
HSP90AB1	1607.859991	0.25570033	69
SIRT1	2215.048411	0.25737705	68
APP	3729.932474	0.25632653	63
KDR	2429.196004	0.25220884	57
ICAM1	1813.331077	0.25445705	56
CYP3A4	2092.784051	0.25160256	55
ACE	2263.673069	0.25322581	54
NR3C1	2162.884329	0.25570033	54
PPARA	2315.896888	0.25160256	52
MAOA	2097.54489	0.25039872	51
MAOB	1518.447362	0.24666143	48
MAPK14	1912.303096	0.24782952	45

**Table 4:** Identified compounds of *M. quinquenervia* leaf essential oil prioritized by degree centrality (DC).

Rank	Name	Score
1	Methyl 2-methylbutyrate	169
2	*m*-Cymene	169
3	*trans*-Verbenol	154
4	*γ*-Terpinene	146
5	Fenchol	143
6	1R-*α*-Pinene	141
7	1S-*α*-Pinene	141
8	Terpinen-4-ol	139
9	Isoborneol	134
10	1,8-Cineole (Eucalyptol)	133
11	(+)-3-Carene	129
12	*α*-Guaiene	127
13	*trans*-Ocimenol	120
14	(−)-Palustrol	101
15	(−)-Globulol	98
16	Viridiflorol	98
17	Ledol	98
18	Caryophyllene oxide	97
19	(+)-Ledene	96

**Table 5:** Interaction details of ligands with amino acid residues at target active sites.

Ligand/Protein	Interacted Amino Acids at the Active Site			
	ESR1	CASP3	PPARG	PTGS2
Methyl 2-methylbutyrate	-	-	-	-
*m*-Cymene	- *pi-pi* t-shaped with PHE99. - Alkyl with LEU41, LEU44, LEU45, MET83, LEU86, LEU123. - *pi*-Alkyl with LEU82, LEU86, PHE99.	-	- *pi*-Anion with GLU59. - *pi*-*pi* t-shaped with PHE64. - Alkyl with ILE62. - *pi*-Alkyl with ARG80.	- Alkyl with VAL318, LEU353, MET491, VAL492. - *pi*-Alkyl with PHE48, LEU321, TRP356.
*trans*-Verbenol	- Alkyl with ALA45 (two), LEU82 (two), LEU86, LEU79	- Conventional hydrogen bond with TYR236, TYR238. - Alkyl with LEU240 (two).	- Alkyl with ALA92, ILE96, MET129 (two). - *pi*-Alkyl with PHE26.	- Carbon hydrogen bond with VAL492, GLY495. - Alkyl with VAL318 (two), LEU321 (two), VAL492, ALA496. - *pi*-Alkyl with TYR317.
*γ*-Terpinene	- Alkyl with LEU86. - *pi*-Alkyl with LEU41, ALA45, LEU82, PHE99.	-	- Amide-*pi* stacked with ARG80. - Alkyl with ILE141, MET148. - *pi*-Alkyl withILE62, ILE81.	- Alkyl with VAL318, LEU353, VAL492. - *pi*-Alkyl with LEU321, TYR354, TRP356, VAL492.
Fenchol	- Alkyl with ALA45, LEU82, LEU86.	-	- Conventional hydrogen bond with ARG95. - Alkyl with LEU28, MET129, LEU133.	- Alkyl with LEU321, VAL492, ALA496.
1R-*α*-Pinene	- Alkyl with LEU41, ALA45 (three), LEU79, LEU82, LEU220.	-	- Alkyl with ALA92 (two bond), ILE96 (one bond), MET129 (two), LEU133 (one). - *pi*-Alkyl with PHE26 (one).	- Alkyl with VAL318 (two), LEU321, VAL492, ALA496 (three).
1S-*α*-Pinene	- Alkyl with ALA45 (three), LEU79 (two), LEU82 (three).	- *pi*-Sigma with TYR236. - Alkyl with MET11 and LEU240 (two). - *pi*-Alkyl with TYR236, TYR238.	- Alkyl with ALA92 (two bond), ILE96 (one bond), MET129 (three), LEU133 (one). - *pi*-Alkyl with PHE26 (one).	- Alkyl with VAL318 (two), LEU321 (two), ALA496 (two).
Terpinen-4-ol	- Alkyl with LEU41, MET116, ILE119. - *pi*-Alkyl with PHE99.	- Conventional hydrogen bond with SER8. - Carbon hydrogen bond with SER8. - *pi*-Sigma with TYR236. - Alkyl with LEU240. - *pi*-Alkyl with TYR236, TYR238.	- Carbon hydrogen bond with GLY84, ILE81. - Unfavorable donor-donor with CYS85. - Alkyl with ILE62, ARG88, ILE141(two).	- Carbon hydrogen bond with VAL492. - Alkyl with LEU321, LEU353, VAL492. - *pi*-Alkyl with TRP354, TRP356.
Isoborneol	- Alkyl with LEU41, ALA45. - *pi*-Alkyl with PHE99.	-	- Alkyl with ALA92, MET129, LEU133.	- Alkyl with LEU321, VAL492, ALA496. - *pi*-Alkyl with PHE487.
1,8-Cineole	- Alkyl with ALA45.	- Conventional hydrogen bond with SER8. - Alkyl with LEU240.	- Alkyl with ALA92, MET129, LEU133.	- Carbon hydrogen bond with ALA496. - Alkyl with VAL318, LEU321, ALA496.

## Data Availability

All data and materials used are available in the manuscript.

## Supplementary Materials

The following supporting information can be downloaded at https://www.mdpi.com/article/10.3390/ph18060771/s1, Figure S1: Chemical structures of the identified components in *M. quinquenervia* leaf essential oil.; Figure S2: 3D and 2D representations of the interaction complexes of ligand-ESR1 interactions.; Figure S3: 3D and 2D representations of the interaction complexes of ligand-CASP3 interactions.; Figure S4: 3D and 2D representations of the interaction complexes of ligand-PPARG interactions.; Figure S5: 3D and 2D representations of the interaction complexes of ligand-PTGS2 interactions.; Figure S6: Dose–response curves illustrating the viability of Vero, MCF-7, HepG-2, and A-549 cell lines following exposure to various concentrations of *M. quinquenervia* leaf essential oil.; Table S1: Pharmacokinetic profiles and drug-likeness parameters of MQLEO bioactive components.; Table S2: Molecular targets of MQLEO bioactive constituents; Table S3: Therapeutic molecular targets implicated in lung cancer pathogenesis.; Table S4: Intersecting targets between MQLEO and lung cancer; Table S5: Topological parameters of PPI network nodes.; Table S6: Protein targets identified after the initial filtration step along with their topological characteristics.; Table S7: Detailed GO enrichment analysis of biological processes.; Table S8: Detailed GO enrichment analysis of cellular components; Table S9: Detailed GO enrichment analysis of molecular functions; Table S10: Comprehensive results of KEGG pathway enrichment analysis.; Table S11: Target proteins, their corresponding grid coordinates, and the amino acid residues of their active sites.

## Abbreviations

The following abbreviations are used in this manuscript:

A-549	Human Lung Cancer Cell Line
ATCC	American Type Culture Collection
BC	Betweenness Centrality
BP	Biological Process
CASP3	Caspase-3
CASTp	Computed Atlas of Surface Topography of Proteins
CC	Closeness Centrality/Cellular Component
CC_50_	50% Cytotoxic Concentration
CDK	Cyclin-Dependent Kinase
CO_2_	Carbon Dioxide
DAVID	Database for Annotation, Visualization, and Integrated Discovery
DMEM	Dulbecco’s Modified Eagle Medium
DMSO	Dimethyl Sulfoxide
eV	Electron Volt
EO	Essential Oil
ESR1	Estrogen Receptor 1 (a nuclear receptor involved in hormone signaling)
FBS	Fetal Bovine Serum
FITC	Fluorescein Isothiocyanate
G_0_-G_1_ Phase	Gap 0/Growth 1 Phase of the Cell Cycle
GC-MS	Gas Chromatography–Mass Spectrometry
GO	Gene Ontology
HepG-2	Human Liver Cancer Cell Line
IC_25_	25% Inhibitory Concentration
IC_50_	Half-maximal Inhibitory Concentration
IL-17	Interleukin 17
kcal/mol	Kilocalories per Mole
KEGG	Kyoto Encyclopedia of Genes and Genomes
MAPK/ERK	Mitogen-Activated Protein Kinase/Extracellular Signal-Regulated Kinase
MCF-7	Human Breast Cancer Cell Line
MF	Molecular Function
MMP	Matrix Metalloproteinase
MQLEO	*Melaleuca quinquenervia* Leaf Essential Oil
MS	Mass Spectrometry
mA	Milliampere
m/z	Mass-to-Charge Ratio
MTT	3-(4,5-Dimethylthiazol-2-yl)-2,5-Diphenyltetrazolium Bromide
NCI	National Cancer Institute
NO	Nitric Oxide
NSCLC	Non-Small Cell Lung Cancer
OD	Optical Density
OMIM	Online Mendelian Inheritance in Man
PDB	Protein Data Bank
PBS	Phosphate-Buffered Saline
PI	Propidium Iodide
PPARG	Peroxisome Proliferator-Activated Receptor Gamma
PPI	Protein–Protein Interaction
PS	Phosphatidylserine
PTGS2	Prostaglandin-Endoperoxide Synthase 2 (COX-2)
RI	Retention Index
RI_Exp._	Experimental Retention Index
RI_Lit._	Literature Retention Index
RMSD	Root Mean Square Deviation
RNase A	Ribonuclease A
RPM	Revolutions Per Minute
Rt	Retention Time In Minutes
S Phase	DNA Synthesis Phase of the Cell Cycle
SD	Standard Deviation
SI	Selectivity Index
STP	Swiss Target Prediction
TNF	Tumor Necrosis Factor
VERO	Normal African Green Monkey Kidney Cells

## References

[1] Pina-Sanchez P., Chavez-Gonzalez A., Ruiz-Tachiquin M., Vadillo E., Monroy-Garcia A., Montesinos J.J., Grajales R., Gutierrez de la Barrera M., Mayani H. (2021). Cancer biology, epidemiology, and treatment in the 21st century: Current status and future challenges from a biomedical perspective. Cancer Control.

[2] Ghosh S., Das S.K., Sinha K., Ghosh B., Sen K., Ghosh N., Sil P.C. (2024). The Emerging Role of Natural Products in Cancer Treatment. Arch. Toxicol..

[3] Wang X., Decker C.C., Zechner L., Krstin S., Wink M. (2019). In vitro wound healing of tumor cells: Inhibition of cell migration by selected cytotoxic alkaloids. BMC Pharmacol. Toxicol..

[4] Niculescu A.-G., Georgescu M., Marinas I.C., Ustundag C.B., Bertesteanu G., Pinteală M., Maier S.S., Al-Matarneh C.M., Angheloiu M., Chifiriuc M.C. (2024). Therapeutic management of malignant wounds: An update. Curr. Treat. Options Oncol..

[5] Tian X., Srinivasan P.R., Tajiknia V., Uruchurtu A.F.S.S., Seyhan A.A., Carneiro B.A., De La Cruz A., Pinho-Schwermann M., George A., Zhao S. (2024). Targeting apoptotic pathways for cancer therapy. J. Clin. Investig..

[6] Wong R.S. (2011). Apoptosis in cancer: From pathogenesis to treatment. J. Exp. Clin. Cancer Res..

[7] Bai J., Li Y., Zhang G. (2017). Cell cycle regulation and anticancer drug discovery. Cancer Biol. Med..

[8] Li C.-X., Wang J.-S., Wang W.-N., Xu D.-K., Zhou Y.-T., Sun F.-Z., Li Y.-Q., Guo F.-Z., Ma J.-L., Zhang X.-Y. (2022). Expression dynamics of periodic transcripts during cancer cell cycle progression and their correlation with anticancer drug sensitivity. Mil. Med. Res..

[9] Sharma M., Grewal K., Jandrotia R., Batish D.R., Singh H.P., Kohli R.K. (2022). Essential oils as anticancer agents: Potential role in malignancies, drug delivery mechanisms, and immune system enhancement. Biomed. Pharmacother..

[10] Wilson P.G. (2010). Myrtaceae. Flowering Plants. Eudicots: Sapindales, Cucurbitales, Myrtaceae.

[11] Barbosa L.C.A., Silva C.J., Teixeira R.R., Meira R.M.S.A., Pinheiro A.L. (2013). Chemistry and biological activities of essential oils from *Melaleuca* L. species. Agric. Conspec. Sci..

[12] Tran P.H., Vu T.T.T., Phan T.D.T., Nguyen V.M., Ngo T.N.M., Le C.V.C., Ton T.H.D. (2024). Chemical compositions and biological properties of the leaf essential oil of three *Melaleuca* species. World Acad. Sci. J..

[13] Liu X., Zu Y., Fu Y., Yao L., Gu C., Wang W., Efferth T. (2009). Antimicrobial activity and cytotoxicity towards cancer cells of *Melaleuca alternifolia* (tea tree) oil. Eur. Food Res. Technol..

[14] Acha E., Ahounou Aikpe J., Adovelande J., Assogba M.F., Agossou G., Sezan A., Dansou H.P., Gbenou J. (2019). Anti-inflammatory properties of *Melaleuca quinquenervia* (Cav.) ST Blake Myrtaceae (Niaouli) leaves’ essential oil. Int. J. Curr. Res. Chem. Pharm. Sci..

[15] Joshi A., Prakash O., Pant A.K., Kumar R., Szczepaniak L., Kucharska-Ambrożej K. (2022). Methyl eugenol, 1, 8-cineole and nerolidol rich essential oils with their biological activities from three *Melaleuca* species growing in Tarai region of North India. Braz. Arch. Biol. Technol..

[16] Monzote L., Scherbakov A.M., Scull R., Satyal P., Cos P., Shchekotikhin A.E., Gille L., Setzer W.N. (2020). Essential oil from *Melaleuca leucadendra*: Antimicrobial, antikinetoplastid, antiproliferative and cytotoxic assessment. Molecules.

[17] Bhagat M., Sangral M., Pandita S., Gupta S., Bindu K. (2017). Pleiotropic chemodiversity in extracts and Essential oil of *Melaleuca viminalis* and *Melaleuca armillaris* of Myrtaceae Family. J. Explor. Res. Pharmacol..

[18] Albayrak İ.G., Gültekin S.K., Konuk M. (2023). Effect of Melaleuca alternifolia oil on cytotoxicity and neuropeptide y gene expression. İstanbul J. Pharm..

[19] Byahatti S., Bogar C., Bhat K., Dandagi G. (2018). Evaluation of anticancer activity of Melaleuka Alternifolia.(ie tea tree oil) on Breast cancer cell line (MDA MB)-An invitro study. Int. J. Med. Microbiol. Trop. Dis..

[20] Chabir N., Romdhane M., Valentin A., Moukarzel B., Marzoug H.N.B., Brahim N.B., Mars M., Bouajila J. (2011). Chemical study and antimalarial, antioxidant, and anticancer activities of *Melaleuca armillaris* (Sol Ex Gateau) Sm essential oil. J. Med. Food.

[21] Govaerts R., Sobral M., Ashton P., Barrie F., Holst B.K., Landrum L.L., Matsumoto K., Mazine F.F., Lughadha E.N., Proneça C. (2008). World Checklist of Myrtaceae.

[22] Ireland B., Hibbert D., Goldsack R., Doran J., Brophy J. (2002). Chemical variation in the leaf essential oil of *Melaleuca quinquenervia* (Cav.) ST Blake. Biochem. Syst. Ecol..

[23] Chaverri C., F Cicció J. (2021). Chemical composition of essential oils of the tree *Melaleuca quinquenervia* (Myrtaceae) cultivated in Costa Rica. Cuad. Investig. UNED.

[24] Aboutabl E., Tohamy S.E., De Footer H., De Buyck L. (1991). A comparative study of the essential oils from three *Melaleuca* species growing in Egypt. Flavour Fragr. J..

[25] Luro F., Garcia Neves C., Costantino G., da Silva Gesteira A., Paoli M., Ollitrault P., Tomi F., Micheli F., Gibernau M. (2020). Effect of Environmental Conditions on the Yield of Peel and Composition of Essential Oils from Citrus Cultivated in Bahia (Brazil) and Corsica (France). Agronomy.

[26] Dakhlaoui S., Bourgou S., Zar Kalai F., Bachkouel S., Ippolito N.M., Msaada K. (2024). Eucalyptus essential oils: Chemical profiling and pharmacological potential for sustainable forest cultivation. Euro-Mediterr. J. Environ. Integr..

[27] Waseem R., Low K.H. (2015). Advanced analytical techniques for the extraction and characterization of plant-derived essential oils by gas chromatography with mass spectrometry. J. Sep. Sci..

[28] Singh M.K., Singh S., Mishra S., Shankar U., Maurya A., Verma R.S., Kumar L., Bharadvaja N., Singh R., Anand R. (2024). Recent Advances in Extraction, Analysis, Value Addition, and Applications of Essential Oils. Medicinal and Aromatic Plants: Current Research Status, Value-Addition to Their Waste, and Agro-Industrial Potential (Volome I).

[29] Chao W.-W., Su C.-C., Peng H.-Y., Chou S.-T. (2017). *Melaleuca quinquenervia* essential oil inhibits α-melanocyte-stimulating hormone-induced melanin production and oxidative stress in B16 melanoma cells. Phytomedicine.

[30] Nishida A., Andoh A. (2025). The Role of Inflammation in Cancer: Mechanisms of Tumor Initiation, Progression, and Metastasis. Cells.

[31] Fernald K., Kurokawa M. (2013). Evading apoptosis in cancer. Trends Cell Biol..

[32] Reuter S., Gupta S.C., Chaturvedi M.M., Aggarwal B.B. (2010). Oxidative stress, inflammation, and cancer: How are they linked?. Free Radic. Biol. Med..

[33] Takeoka G.R., Buttery R.G., Flath R.A. (1992). Volatile constituents of Asian pear (Pyrus serotina). J. Agric. Food Chem..

[34] Carasek E., Pawliszyn J. (2006). Screening of tropical fruit volatile compounds using solid-phase microextraction (SPME) fibers and internally cooled SPME fiber. J. Agric. Food Chem..

[35] Hoskovec M., Grygarová D., Cvačka J., Streinz L., Zima J., Verevkin S.P., Koutek B. (2005). Determining the vapour pressures of plant volatiles from gas chromatographic retention data. J. Chromatogr. A.

[36] Fikry E., Orfali R., Tawfeek N., Perveen S., Ghafar S., El-Domiaty M.M., El-Shafae A.M. (2024). Unveiling the Bioactive Efficacy of Cupressus sempervirens ‘Stricta’ Essential Oil: Composition, In Vitro Activities, and In Silico Analyses. Pharmaceuticals.

[37] Allegrone G., Belliardo F., Cabella P. (2006). Comparison of Volatile Concentrations in Hand-Squeezed Juices of Four Different Lemon Varieties. J. Agric. Food Chem..

[38] Medina A.L., Lucero M.E., Holguin F.O., Estell R.E., Posakony J.J., Simon J., O’Connell M.A. (2005). Composition and antimicrobial activity of *Anemopsis californica* leaf oil. J. Agric. Food Chem..

[39] Siani A.C., Garrido I.S., Monteiro S.S., Carvalho E.S., Ramos M.F.S. (2004). *Protium icicariba* as a source of volatile essences. Biochem. Syst. Ecol..

[40] Paolini J., Muselli A., Bernardini A.-F., Bighelli A., Casanova J., Costa J. (2007). Thymol derivatives from essential oil of *Doronicum corsicum* L.. Flavour Fragr. J..

[41] Hazzit M., Baaliouamer A., Faleiro M.L., Miguel M.G. (2006). Composition of the Essential Oils of Thymus and Origanum Species from Algeria and Their Antioxidant and Antimicrobial Activities. J. Agric. Food Chem..

[42] Tayoub G., Schwob I., Bessière J.-M., Masotti V., Rabier J., Ruzzier M., Viano J. (2006). Composition of volatile oils of Styrax (*Styrax officinalis* L.) leaves at different phenological stages. Biochem. Syst. Ecol..

[43] Fikry E., Orfali R., Elbaramawi S.S., Perveen S., El-Shafae A.M., El-Domiaty M.M., Tawfeek N. (2023). *Chamaecyparis lawsoniana* leaf essential oil as a potential anticancer agent: Experimental and computational studies. Plants.

[44] Mohanty D., Padhee S., Priyadarshini A., Champati B.B., Das P.K., Jena S., Sahoo A., Panda P.C., Nayak S., Ray A. (2024). Elucidating the anti-cancer potential of *Cinnamomum tamala* essential oil against non-small cell lung cancer: A multifaceted approach involving GC-MS profiling, network pharmacology, and molecular dynamics simulations. Heliyon.

[45] Zhang Y., Tang J., Liu Q., Ge J., Ma Z., Mou J., Wang L. (2023). Biological, functional and network pharmacological exploration of essential oils in treatment and healthcare of human diseases. Future Integr. Med..

[46] Olmedo R.H., Asensio C.M., Grosso N.R. (2015). Thermal stability and antioxidant activity of essential oils from aromatic plants farmed in Argentina. Ind. Crops Prod..

[47] Abdelmohsen U.R., Elmaidomy A.H. (2025). Exploring the therapeutic potential of essential oils: A review of composition and influencing factors. Front. Nat. Prod..

[48] Turek C., Stintzing F.C. (2013). Stability of essential oils: A review. Compr. Rev. Food Sci. Food Saf..

[49] Mahanta B.P., Bora P.K., Kemprai P., Borah G., Lal M., Haldar S. (2021). Thermolabile essential oils, aromas and flavours: Degradation pathways, effect of thermal processing and alteration of sensory quality. Food Res. Int..

[50] Barra A. (2009). Factors affecting chemical variability of essential oils: A review of recent developments. Nat. Prod. Commun..

[51] Kocabaş Oğuz I., Kaplan M. (2023). The effect of altitude and soil properties on the essential oil components of Turkish sage (*Salvia fruticosa* Mill.). Bol. Latinoam. Caribe Plantas Med. Aromát..

[52] Malsy M., Bitzinger D., Graf B., Bundscherer A. (2019). Staurosporine induces apoptosis in pancreatic carcinoma cells PaTu 8988t and Panc-1 via the intrinsic signaling pathway. Eur. J. Med. Res..

[53] Montalvão M.M., Felix F.B., Propheta Dos Santos E.W., Santos J.F., de Lucca Júnior W., Farias A.S., de Souza Ribeiro A., Cavaleiro C., Machado S.M.F., Scher R. (2023). Cytotoxic activity of essential oil from Leaves of Myrcia splendens against A549 Lung Cancer cells. BMC Complement. Med. Ther..

[54] Alqudah M.A., Yaseen M.M., Alzoubi K.H., Al-Husein B.A., Bardaweel S.K., Abuhelwa A.Y., Semreen A.M., Zenati R.A., El-Awady R., Shara M. (2025). Metabolomic Analysis, Antiproliferative, Anti-Migratory, and Anti-Invasive Potential of Amlodipine in Lung Cancer Cells. Drug Des. Dev. Ther..

[55] Liew S.K., Azmi M.N., In L.L., Awang K., Nagoor N.H. (2017). Anti-proliferative, apoptotic induction, and anti-migration effects of hemi-synthetic 1′ S-1′-acetoxychavicol acetate analogs on MDA-MB-231 breast cancer cells. Drug Des. Dev. Ther..

[56] Wang L., Ruan M., Bu Q., Zhao C. (2025). Signaling Pathways Driving MSC Osteogenesis: Mechanisms, Regulation, and Translational Applications. Int. J. Mol. Sci..

[57] Selvi H., Brüning-Richardson A., Danovi D. (2025). Systematic Review of Pre-Clinical Systems Using Artificial Microenvironments and Anti-Migratory Drugs to Control Migration of Glioblastoma Cells. Expert Rev. Mol. Med..

[58] Maltese W.A., Overmeyer J.H. (2015). Non-apoptotic cell death associated with perturbations of macropinocytosis. Front. Physiol..

[59] Lataster L., Huber H.M., Böttcher C., Föller S., Takors R., Radziwill G. (2023). Cell Cycle Control by Optogenetically Regulated Cell Cycle Inhibitor Protein p21. Biology.

[60] Lossaint G., Horvat A., Gire V., Bačević K., Mrouj K., Charrier-Savournin F., Georget V., Fisher D., Dulić V. (2022). Reciprocal regulation of p21 and Chk1 controls the cyclin D1-RB pathway to mediate senescence onset after G2 arrest. J. Cell Sci..

[61] Gandhi D.S., Gupta I., Menia R., Kumar R., Kara A., Gelen V., Kara H. (2023). Perspective Chapter: Molecular Pathology of Lung Cancer. Molecular Histopathology and Cytopathology.

[62] Hsu R., Leyba A., Chen D., Krause H.B., Elliott A., Cozen W., Roussos Torres E.T., Nagasaka M., Mamdani H., Lopes G. (2024). Survival and mutational differences based on ESR1 and ESR2 expression in non-small cell lung cancer (NSCLC). J. Clin. Oncol..

[63] Hamilton D.H., Griner L.M., Keller J.M., Hu X., Southall N., Marugan J., David J.M., Ferrer M., Palena C. (2016). Targeting estrogen receptor signaling with fulvestrant enhances immune and chemotherapy-mediated cytotoxicity of human lung cancer. Clin. Cancer Res..

[64] Li Q., Li Z., Luo T., Shi H. (2022). Targeting the PI3K/AKT/mTOR and RAF/MEK/ERK pathways for cancer therapy. Mol. Biomed..

[65] Eskandari E., Negri G.L., Tan S., MacAldaz M.E., Ding S., Long J., Nielsen K., Spencer S.E., Morin G.B., Eaves C.J. (2024). Dependence of human cell survival and proliferation on the CASP3 prodomain. Cell Death Discov..

[66] Eskandari E., Eaves C.J. (2022). Paradoxical roles of caspase-3 in regulating cell survival, proliferation, and tumorigenesis. J. Cell Biol..

[67] Gökhan A. (2022). Evaluation of cytotoxic, membrane damaging and apoptotic effects of *Origanum majorana* essential oil on lung cancer and epidermoid carcinoma cells. Evaluation.

[68] Saini D., Chaudhary P.K., Chaudhary J.K., Kaur H., Verma G.K., Pramanik S.D., Roy P., Mirza-Shariff A.A., Prasad R. (2024). Molecular mechanisms of antiproliferative and pro-apoptotic effects of essential oil active constituents in MCF7 and T24 cancer cell lines: In vitro insights and in silico modelling of proapoptotic gene product-compound interactions. Apoptosis.

[69] Zhang J., Tang M., Shang J. (2024). PPARγ Modulators in Lung Cancer: Molecular Mechanisms, Clinical Prospects, and Challenges. Biomolecules.

[70] Xu R., Luo X., Ye X., Li H., Liu H., Du Q., Zhai Q. (2021). SIRT1/PGC-1α/PPAR-γ correlate with hypoxia-induced chemoresistance in non-small cell lung cancer. Front. Oncol..

[71] Lin X.-M., Luo W., Wang H., Li R.-Z., Huang Y.-S., Chen L.-K., Wu X.-P. (2019). The Role of Prostaglandin-Endoperoxide Synthase-2 in Chemoresistance of Non-Small Cell Lung Cancer. Front. Pharmacol..

[72] Lai H., Liu Y., Wu J., Cai J., Jie H., Xu Y., Deng S. (2022). Targeting cancer-related inflammation with non-steroidal anti-inflammatory drugs: Perspectives in pharmacogenomics. Front. Pharmacol..

[73] Wang D., Xia D., DuBois R.N. (2011). The crosstalk of PTGS2 and EGF signaling pathways in colorectal cancer. Cancers.

[74] Seol G.H., Kim K.Y. (2016). Eucalyptol and its role in chronic diseases. Drug Discov. Mother Nat..

[75] Singh P.P., Jaiswal A.K., Kumar A., Gupta V., Prakash B. (2021). Untangling the multi-regime molecular mechanism of verbenol-chemotype Zingiber officinale essential oil against Aspergillus flavus and aflatoxin B1. Sci. Rep..

[76] Paduch R., Trytek M., Król S.K., Kud J., Frant M., Kandefer-Szerszeń M., Fiedurek J. (2016). Biological activity of terpene compounds produced by biotechnological methods. Pharm. Biol..

[77] Wojtunik-Kulesza K.A., Cieśla Ł.M., Waksmundzka-Hajnos M. (2018). Approach to determination a structure–antioxidant activity relationship of selected common terpenoids evaluated by ABTS•+ radical cation assay. Nat. Prod. Commun..

[78] Salehi B., Upadhyay S., Erdogan Orhan I., Kumar Jugran A., L.D. Jayaweera S., A. Dias D., Sharopov F., Taheri Y., Martins N., Baghalpour N. (2019). Therapeutic Potential of α- and β-Pinene: A Miracle Gift of Nature. Biomolecules.

[79] Deen J.I., Zawad A.S., Uddin M., Chowdhury M.A.H., Al Araby S.Q., Rahman M.A. (2023). Terpinen-4-ol, A volatile terpene molecule, extensively electrifies the biological systems against the oxidative stress-linked pathogenesis. Adv. Redox Res..

[80] Yadav N., Chandra H. (2017). Suppression of inflammatory and infection responses in lung macrophages by eucalyptus oil and its constituent 1, 8-cineole: Role of pattern recognition receptors TREM-1 and NLRP3, the MAP kinase regulator MKP-1, and NFκB. PLoS ONE.

[81] Gao K., Wu C., Li Y., Lu J., Jiang Y. (2022). Transcriptome Analysis Reveals the Anti-Tumor Mechanism of Eucalyptol Treatment on Neuroblastoma Cell Line SH-SY5Y. Neurochem. Res..

[82] Hu T., Shi R., Xu Y., Xu T., Fang Y., Gu Y., Zhou Z., Shu Y. (2025). Multi-omics and single-cell analysis reveals machine learning-based pyrimidine metabolism-related signature in the prognosis of patients with lung adenocarcinoma. Int. J. Med. Sci..

[83] Zhang N., Zhang T., Liu J., Sun R., Yu Z., Shen G. (2024). Exploring the Mechanism of Comfrey in Lung Cancer Based on Network Pharmacology and Molecular Docking. SAS J. Med..

[84] Pervin S., Singh R., Sen S., Chaudhuri G. (2010). Dual role of nitric oxide in cancer biology. Nitric Oxide (NO) and Cancer: Prognosis, Prevention, and Therapy.

[85] Lee C.B., Choi H.G., Gurmessa S.K., Jang I.-T., Kumar N., Jiang Z., Kaushik N.K., Kim H.-J. (2024). Enhancing antitumor immunity in Lewis lung cancer through plasma-treated medium-induced activation of dendritic cells. Cancer Cell Int..

[86] Singh P., Lim B. (2022). Targeting apoptosis in cancer. Curr. Oncol. Rep..

[87] Ross K.E., Zhang G., Akcora C., Lin Y., Fang B., Koomen J., Haura E.B., Grimes M. (2023). Network models of protein phosphorylation, acetylation, and ubiquitination connect metabolic and cell signaling pathways in lung cancer. PLoS Comput. Biol..

[88] Zhang B., Leung P.-C., Cho W.C.-S., Wong C.-K., Wang D. (2025). Targeting PI3K signaling in Lung Cancer: Advances, challenges and therapeutic opportunities. J. Transl. Med..

[89] Xia M., Zhang Y., Jin K., Lu Z., Zeng Z., Xiong W. (2019). Communication between mitochondria and other organelles: A brand-new perspective on mitochondria in cancer. Cell Biosci..

[90] Kuo C.-L., Ponneri Babuharisankar A., Lin Y.-C., Lien H.-W., Lo Y.K., Chou H.-Y., Tangeda V., Cheng L.-C., Cheng A.N., Lee A.Y.-L. (2022). Mitochondrial oxidative stress in the tumor microenvironment and cancer immunoescape: Foe or friend?. J. Biomed. Sci..

[91] Zhou Q., Cao T., Li F., Zhang M., Li X., Zhao H., Zhou Y. (2024). Mitochondria: A new intervention target for tumor invasion and metastasis. Mol. Med..

[92] Wang S.-F., Tseng L.-M., Lee H.-C. (2023). Role of mitochondrial alterations in human cancer progression and cancer immunity. J. Biomed. Sci..

[93] Harris J.R., Marles-Wright J. (2022). Macromolecular Protein Complexes IV: Structure and Function.

[94] Korde Choudhari S., Chaudhary M., Bagde S., Gadbail A.R., Joshi V. (2013). Nitric oxide and cancer: A review. World J. Surg. Oncol..

[95] Keshet R., Erez A. (2018). Arginine and the metabolic regulation of nitric oxide synthesis in cancer. Dis. Models Mech..

[96] Martins F., Rosspopoff O., Carlevaro-Fita J., Forey R., Offner S., Planet E., Pulver C., Pak H., Huber F., Michaux J. (2024). A Cluster of Evolutionarily Recent KRAB Zinc Finger Proteins Protects Cancer Cells from Replicative Stress–Induced Inflammation. Cancer Res..

[97] Guo H., Wang S., Zhang H., Li J., Wang C., Liu Z., Chen J., Wang K., Wei X., Wei Q. (2024). Research progress on the molecular structure, function, and application in tumor therapy of zinc transporter ZIP4. Int. J. Biol. Sci..

[98] Zhao L., Gui Y., Cai J., Deng X. (2025). Biometallic ions and derivatives: A new direction for cancer immunotherapy. Mol. Cancer.

[99] Zhang X., Li B., Lan T., Chiari C., Ye X., Wang K., Chen J. (2025). The role of interleukin-17 in inflammation-related cancers. Front. Immunol..

[100] Laha D., Grant R., Mishra P., Nilubol N. (2021). The role of tumor necrosis factor in manipulating the immunological response of tumor microenvironment. Front. Immunol..

[101] Webster J.D., Vucic D. (2020). The balance of TNF mediated pathways regulates inflammatory cell death signaling in healthy and diseased tissues. Front. Cell Dev. Biol..

[102] Ahrens T.D., Bang-Christensen S.R., Jørgensen A.M., Løppke C., Spliid C.B., Sand N.T., Clausen T.M., Salanti A., Agerbæk M.Ø. (2020). The role of proteoglycans in cancer metastasis and circulating tumor cell analysis. Front. Cell Dev. Biol..

[103] Glaviano A., Lau H.S.-H., Carter L.M., Lee E.H.C., Lam H.Y., Okina E., Tan D.J.J., Tan W., Ang H.L., Carbone D. (2025). Harnessing the tumor microenvironment: Targeted cancer therapies through modulation of epithelial-mesenchymal transition. J. Hematol. Oncol..

[104] Amos S.E., Choi Y.S. (2021). The cancer microenvironment: Mechanical challenges of the metastatic cascade. Front. Bioeng. Biotechnol..

[105] Yuan Z., Li Y., Zhang S., Wang X., Dou H., Yu X., Zhang Z., Yang S., Xiao M. (2023). Extracellular matrix remodeling in tumor progression and immune escape: From mechanisms to treatments. Mol. Cancer.

[106] Ravegnini G., Sammarini G., Hrelia P., Angelini S. (2015). Key genetic and epigenetic mechanisms in chemical carcinogenesis. Toxicol. Sci..

[107] Elliott M.R., Koster K.M., Murphy P.S. (2017). Efferocytosis signaling in the regulation of macrophage inflammatory responses. J. Immunol..

[108] Caner A. (2024). Immune Escape Mechanism of Cancer. Curr. Mol. Biol. Rep..

[109] Chatatikun M., Pattaranggoon N.C., Sama-ae I., Ranteh O., Poolpirom M., Pantanakong O., Chumworadet P., Kawakami F., Imai M., Tedasen A. (2024). Mechanistic exploration of bioactive constituents in Gnetum gnemon for GPCR-related cancer treatment through network pharmacology and molecular docking. Sci. Rep..

[110] Cory A.H., Owen T.C., Barltrop J.A., Cory J.G. (1991). Use of an aqueous soluble tetrazolium/formazan assay for cell growth assays in culture. Cancer Commun..

[111] Gomha S.M., Riyadh S.M., Mahmmoud E.A., Elaasser M.M. (2015). Synthesis and anticancer activity of arylazothiazoles and 1,3,4-thiadiazoles using chitosan-grafted-poly(4-vinylpyridine) as a novel copolymer basic catalyst. Chem. Heterocycl. Compd..

[112] Lipinski C.A. (2004). Lead-and drug-like compounds: The rule-of-five revolution. Drug Discov. Today Technol..

[113] Daina A., Michielin O., Zoete V. (2019). SwissTargetPrediction: Updated data and new features for efficient prediction of protein targets of small molecules. Nucleic Acids Res..

[114] Yao Z.J., Dong J., Che Y.J., Zhu M.F., Wen M., Wang N.N., Wang S., Lu A.P., Cao D.S. (2016). TargetNet: A web service for predicting potential drug-target interaction profiling via multi-target SAR models. J. Comput. Aided Mol. Des..

[115] Hamosh A., Scott A.F., Amberger J.S., Bocchini C.A., McKusick V.A. (2005). Online Mendelian Inheritance in Man (OMIM), a knowledgebase of human genes and genetic disorders. Nucleic Acids Res..

[116] Rebhan M., Chalifa-Caspi V., Prilusky J., Lancet D. (1997). GeneCards: Integrating information about genes, proteins and diseases. Trends Genet. TIG.

[117] Safran M., Dalah I., Alexander J., Rosen N., Iny Stein T., Shmoish M., Nativ N., Bahir I., Doniger T., Krug H. (2010). GeneCards Version 3: The human gene integrator. Database.

[118] Zaru R., Orchard S., Consortium U. (2023). Uniprot tools: BLAST, align, peptide search, and ID mapping. Curr. Protoc..

[119] Szklarczyk D., Kirsch R., Koutrouli M., Nastou K., Mehryary F., Hachilif R., Gable A.L., Fang T., Doncheva N.T., Pyysalo S. (2023). The STRING database in 2023: Protein–protein association networks and functional enrichment analyses for any sequenced genome of interest. Nucleic Acids Res..

[120] Shannon P., Markiel A., Ozier O., Baliga N.S., Wang J.T., Ramage D., Amin N., Schwikowski B., Ideker T. (2003). Cytoscape: A software environment for integrated models of biomolecular interaction networks. Genome Res..

[121] Tang Y., Li M., Wang J., Pan Y., Wu F.X. (2015). CytoNCA: A cytoscape plugin for centrality analysis and evaluation of protein interaction networks. Biosystems.

[122] Chin C.-H., Chen S.-H., Wu H.-H., Ho C.-W., Ko M.-T., Lin C.-Y. (2014). cytoHubba: Identifying hub objects and sub-networks from complex interactome. BMC Syst. Biol..

[123] Dennis G., Sherman B.T., Hosack D.A., Yang J., Gao W., Lane H.C., Lempicki R.A. (2003). DAVID: Database for annotation, visualization, and integrated discovery. Genome Biol..

[124] Vajdos F.F., Hoth L.R., Geoghegan K.F., Simons S.P., LeMotte P.K., Danley D.E., Ammirati M.J., Pandit J. (2007). The 2.0 Å crystal structure of the ERα ligand-binding domain complexed with lasofoxifene. Protein Sci..

[125] Maciag J.J., Mackenzie S.H., Tucker M.B., Schipper J.L., Swartz P., Clark A.C. (2016). Tunable allosteric library of caspase-3 identifies coupling between conserved water molecules and conformational selection. Proc. Natl. Acad. Sci. USA.

[126] Arifi S., Marschner J.A., Pollinger J., Isigkeit L., Heitel P., Kaiser A., Obeser L., Höfner G., Proschak E., Knapp S. (2023). Targeting the alternative vitamin E metabolite binding site enables noncanonical PPARγ modulation. J. Am. Chem. Soc..

[127] Lucido M.J., Orlando B.J., Vecchio A.J., Malkowski M.G. (2016). Crystal structure of aspirin-acetylated human cyclooxygenase-2: Insight into the formation of products with reversed stereochemistry. Biochemistry.

[128] Burley S.K., Berman H.M., Kleywegt G.J., Markley J.L., Nakamura H., Velankar S., Wlodawer A., Dauter Z., Jaskolski M. (2017). Protein Data Bank (PDB): The Single Global Macromolecular Structure Archive. Protein Crystallography: Methods and Protocols.

[129] Pettersen E.F., Goddard T.D., Huang C.C., Couch G.S., Greenblatt D.M., Meng E.C., Ferrin T.E. (2004). UCSF Chimera—A visualization system for exploratory research and analysis. J. Comput. Chem..

[130] Fikry E., Orfali R., El-Sayed S.S., Perveen S., Ghafar S., El-Shafae A.M., El-Domiaty M.M., Tawfeek N. (2023). Potential Hepatoprotective Effects of *Chamaecyparis lawsoniana* against Methotrexate-Induced Liver Injury: Integrated Phytochemical Profiling, Target Network Analysis, and Experimental Validation. Antioxidants.

[131] Tian W., Chen C., Lei X., Zhao J., Liang J. (2018). CASTp 3.0: Computed atlas of surface topography of proteins. Nucleic Acids Res..

[132] O’Boyle N.M., Banck M., James C.A., Morley C., Vandermeersch T., Hutchison G.R. (2011). Open Babel: An open chemical toolbox. J. Cheminform..

[133] Biovia D.S. (2021). Discovery Studio Visualizer.

