# Supplementary material for: Chemical Composition and Anti-Lung Cancer Activities of Melaleuca quinquenervia Leaf Essential Oil: Integrating Gas Chromatography–Mass Spectrometry (GC/MS) Profiling, Network Pharmacology, and Molecular Docking
Source: Pharmaceuticals (Basel). 2025 May 22;18(6):771. doi: 10.3390/ph18060771 (PMC12196179; doi:10.3390/ph18060771)
Supplement: Supplementary file 1 [file pharmaceuticals-18-00771-s001.zip › pharmaceuticals-3625015-supplementary/Revised_Supplementary_pharmaceuticals/Figure S1.pdf]

Figure S1. Chemical structures of the identified components in *Melaleuca quinquenervia* leaf essential oil.

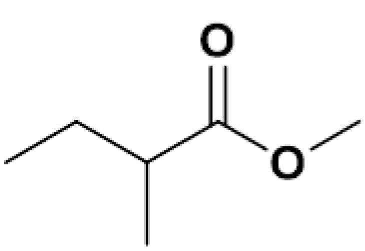

Butanoic acid, 2-methyl-, methyl ester

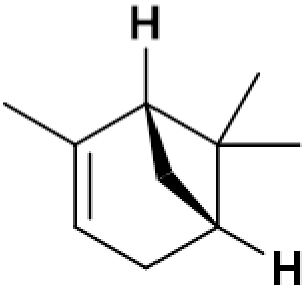

1R-*α*-Pinene

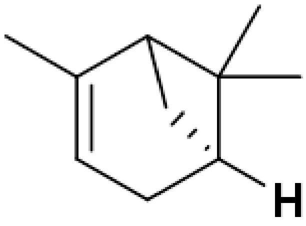

1S-*α*-Pinene

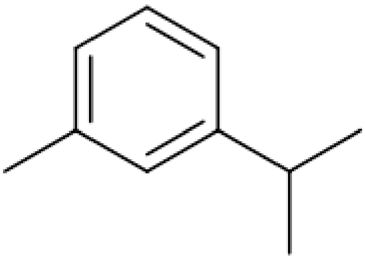

***m*-Cymene**

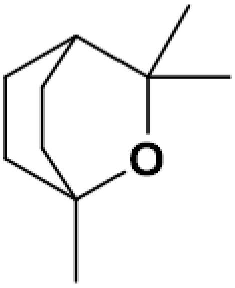

1,8-Cineole (Eucalyptol)

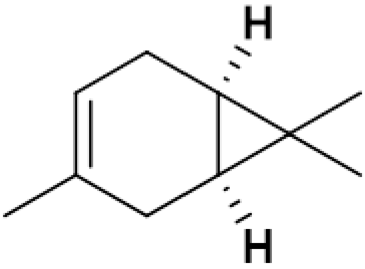

(+)-3-Carene

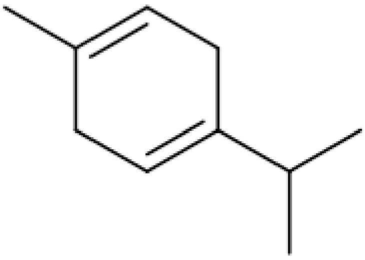

γ-Terpinene

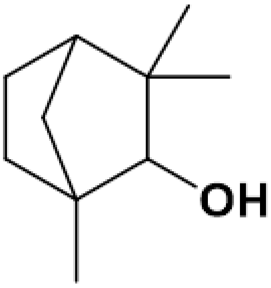

Fenchol

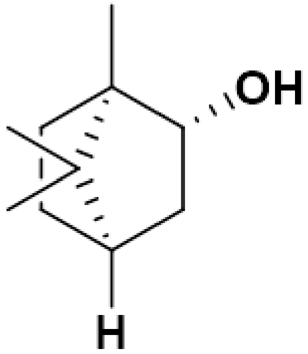

Isoborneol

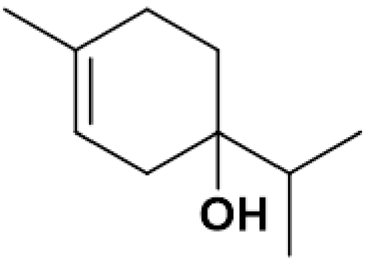

Terpinen-4-ol

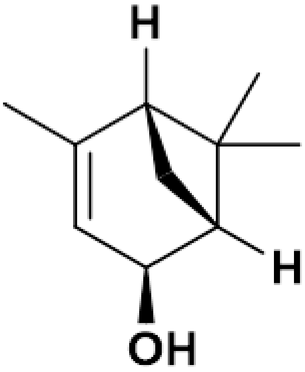

***trans*-Verbenol**

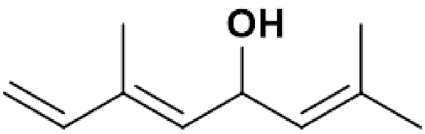

***trans*-Ocimenol**

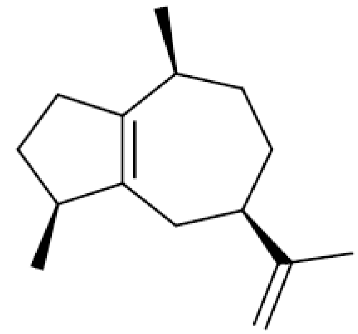

α-Guaiene

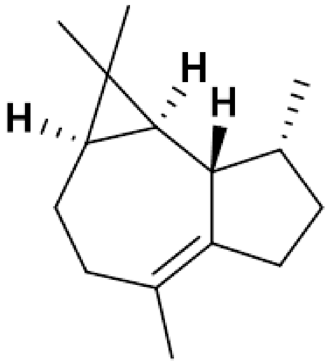

(+)-Ledene

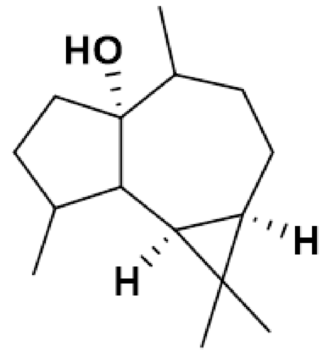

(-)-Palustrol

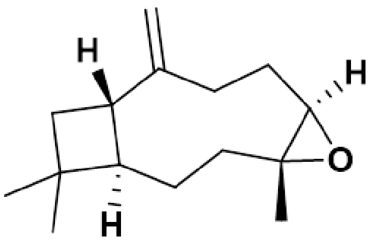

Caryophyllene oxide

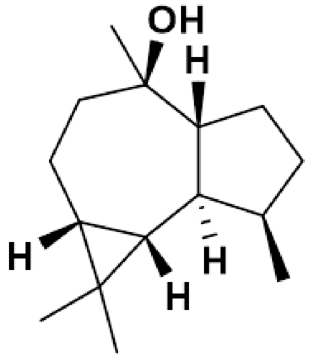

(-)-Globulol

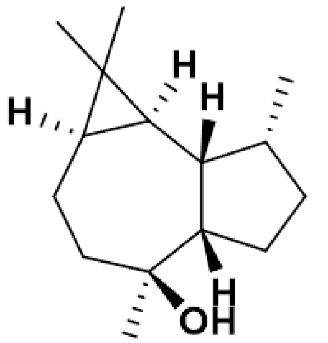

Viridiflorol

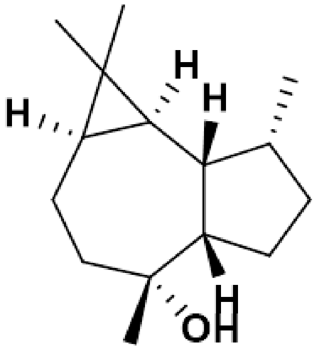

Ledol
