# Supplementary material for: Chemical Composition and Anti-Lung Cancer Activities of Melaleuca quinquenervia Leaf Essential Oil: Integrating Gas Chromatography–Mass Spectrometry (GC/MS) Profiling, Network Pharmacology, and Molecular Docking
Source: Pharmaceuticals (Basel). 2025 May 22;18(6):771. doi: 10.3390/ph18060771 (PMC12196179; doi:10.3390/ph18060771)
Supplement: Supplementary file 1 [file pharmaceuticals-18-00771-s001.zip › pharmaceuticals-3625015-supplementary/Revised_Supplementary_pharmaceuticals/Figure S2-S5.pdf]

ESR1_*m*-Cymene

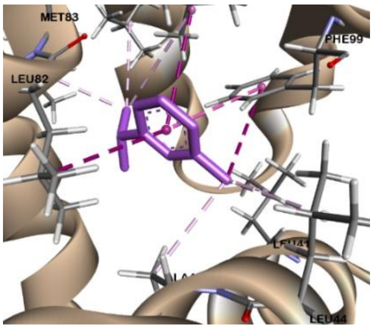

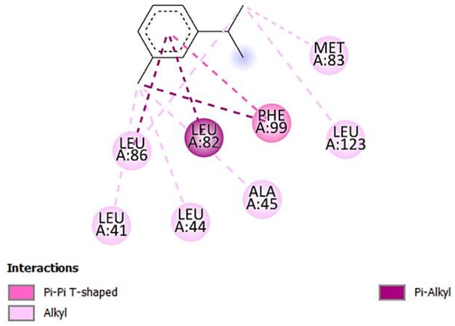

ESR1_*trans*-Verbenol

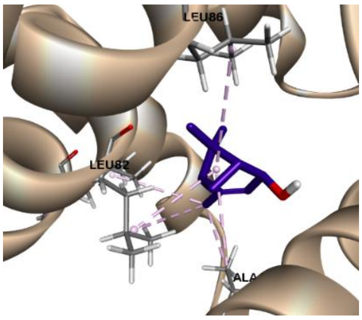

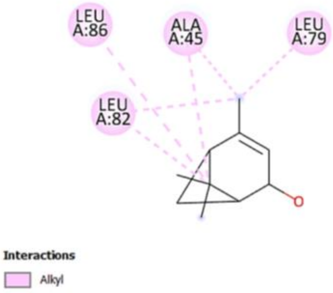

ESR1_*γ*-Terpinene

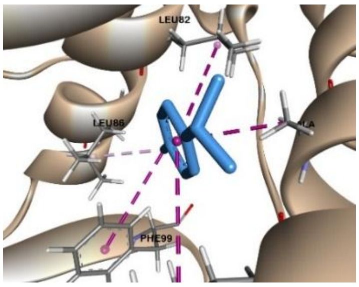

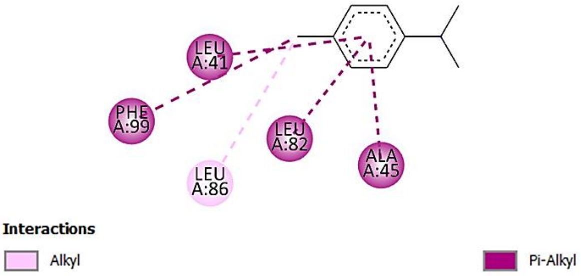

ESR1_Fenchol

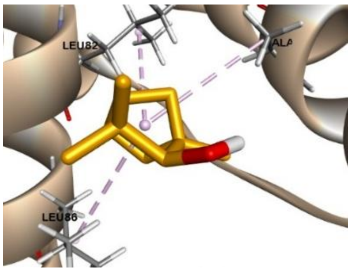

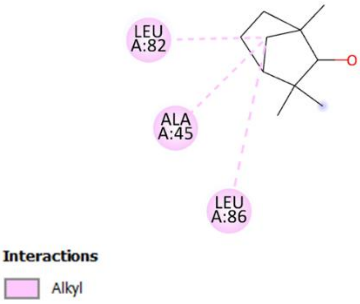

ESR1_1R-*α*-Pinene

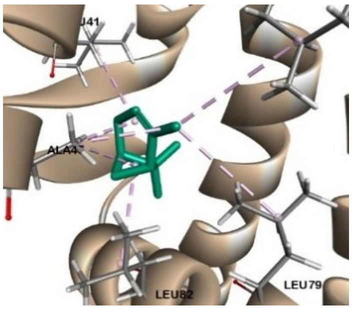

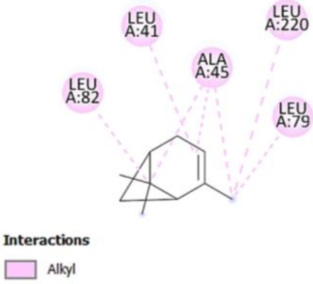

ESR1_1S-*α*-Pinene

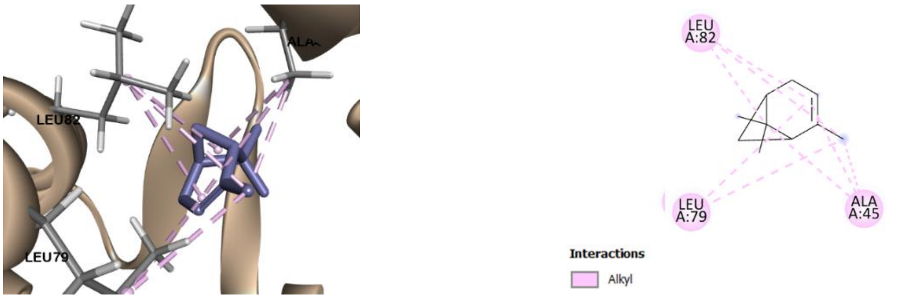

ESR1_Terpinen-4-ol

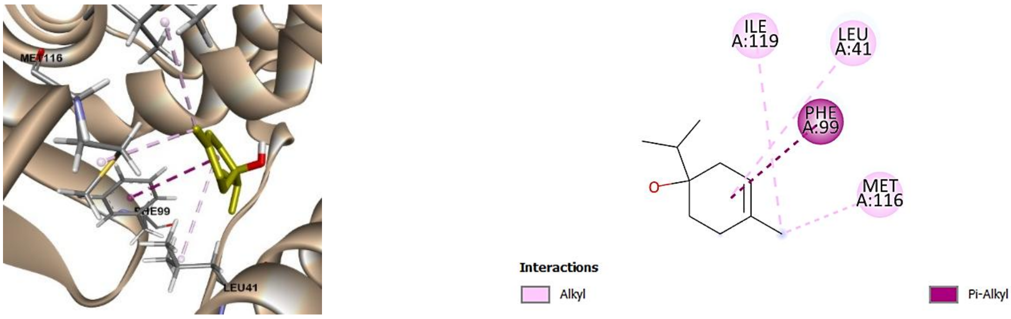

Isoborneol

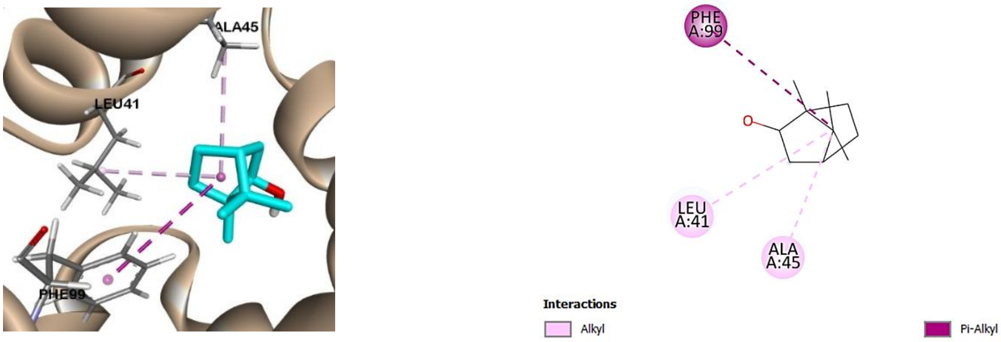

ESR1_1,8-Cineole

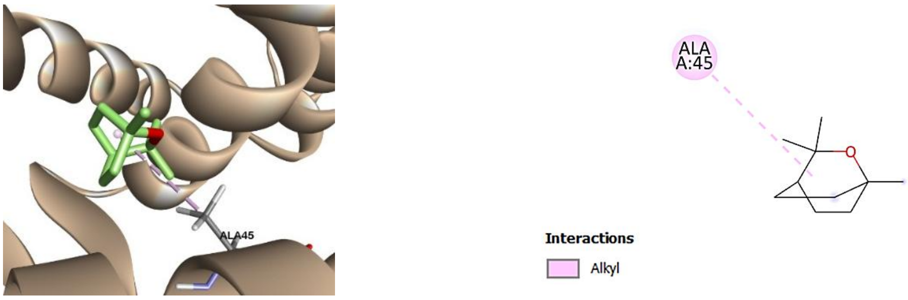

Figure S2. 3D and 2D representations of the interaction complexes of ligand-ESR1 interactions.

CASP3_*trans*-Verbenol

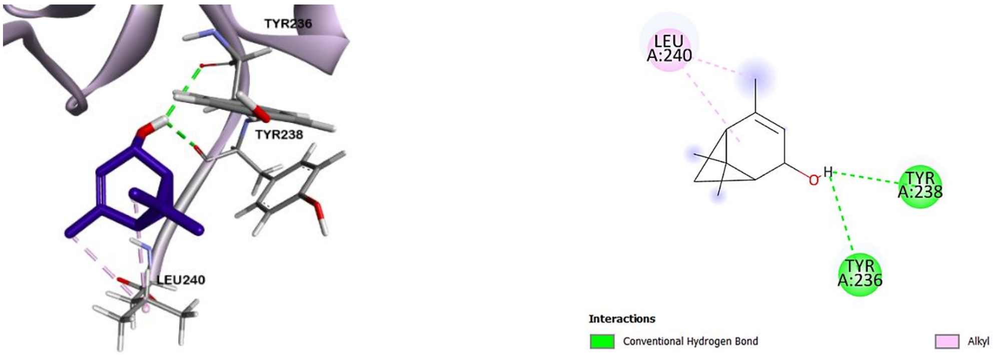

CASP3_1S-*α*-Pinene

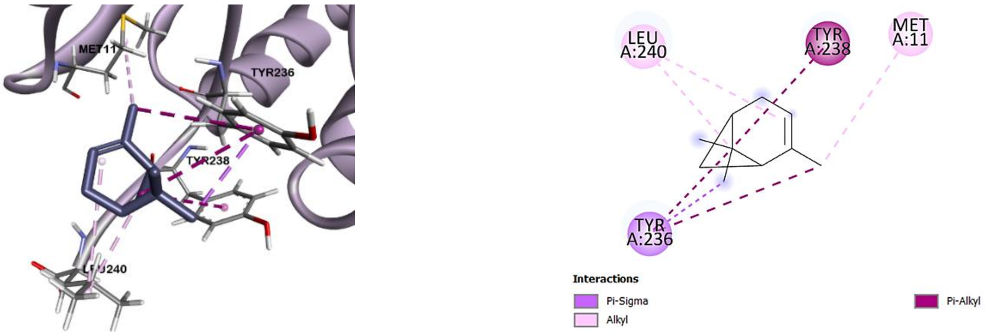

CASP3_Terpinen-4-ol

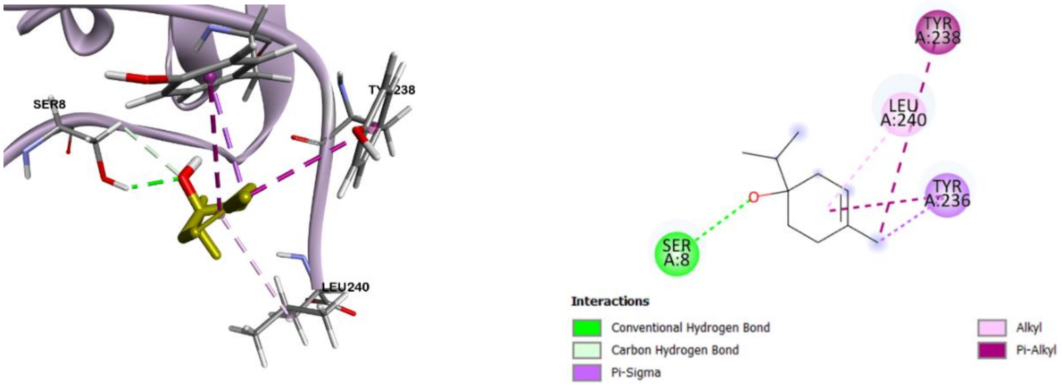

CASP3_1,8-Cineole

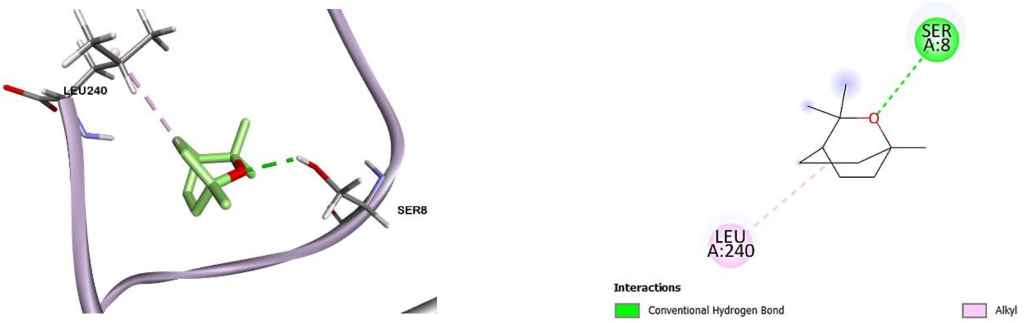

Figure S3. 3D and 2D representations of the interaction complexes of ligand-CASP3 interactions.

PPARG_*m*-Cymene

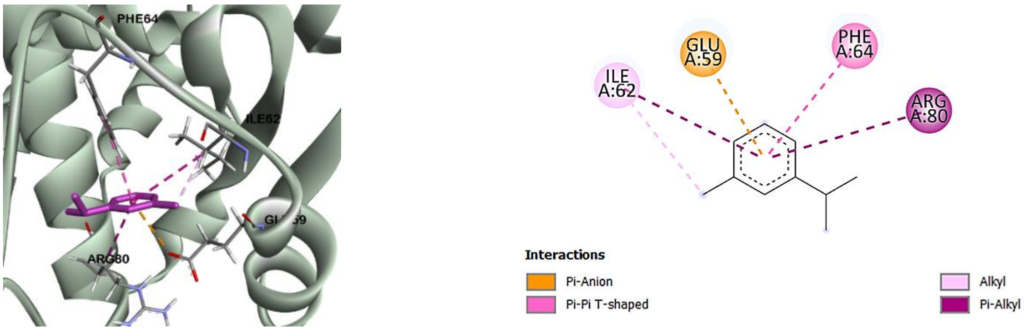

PPARG_*trans*-Verbenol

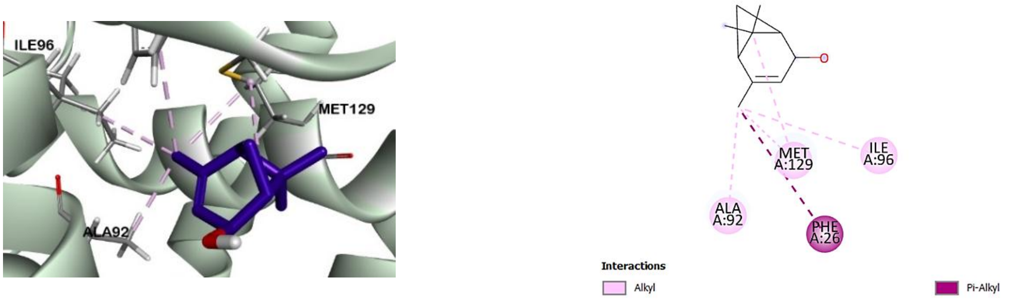

PPARG_γ-Terpinene

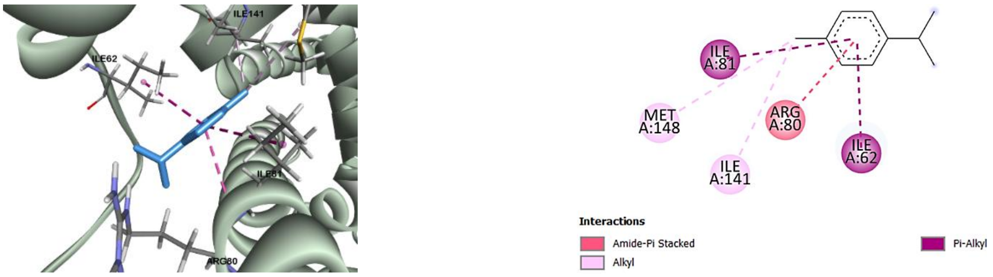

PPARG_Fenchol

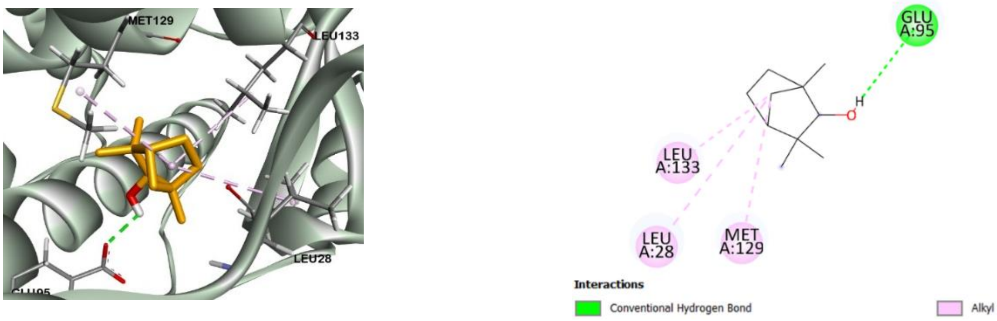

PPARG_1R-*α*-Pinene

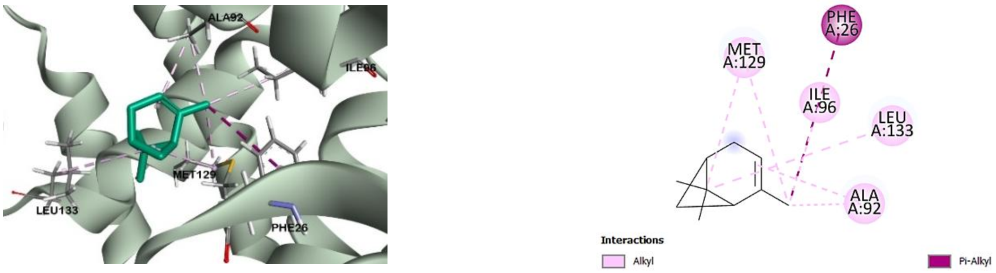

PPARG_1S-α-Pinene

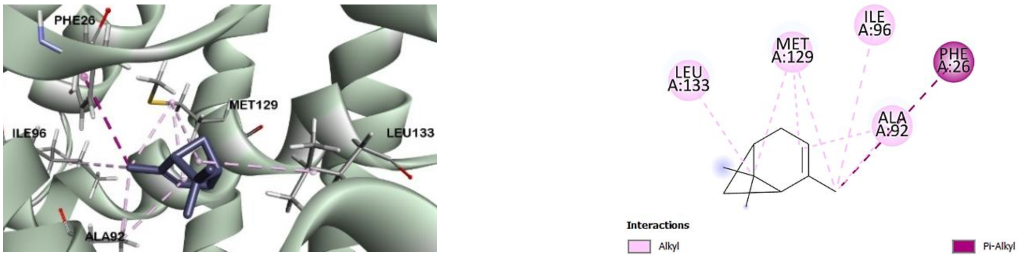

PPARG_Terpinen-4-ol

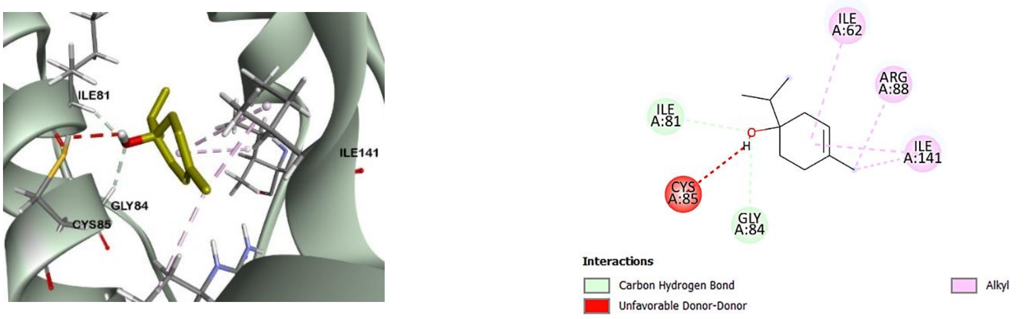

PPARG_Isoborneol

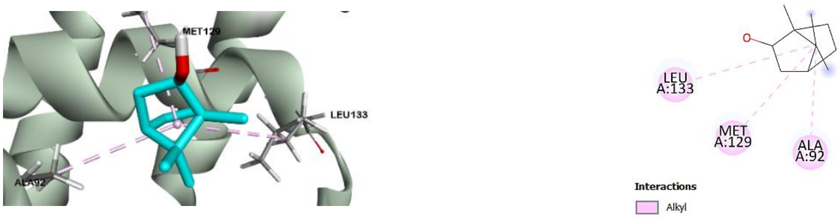

PPARG_1,8-Cineole

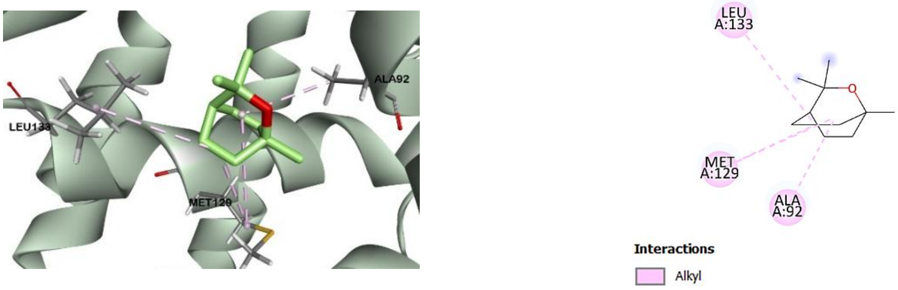

Figure S4. 3D and 2D representations of the interaction complexes of ligand-PPARG interactions.

PTGS2_*m*-Cymene

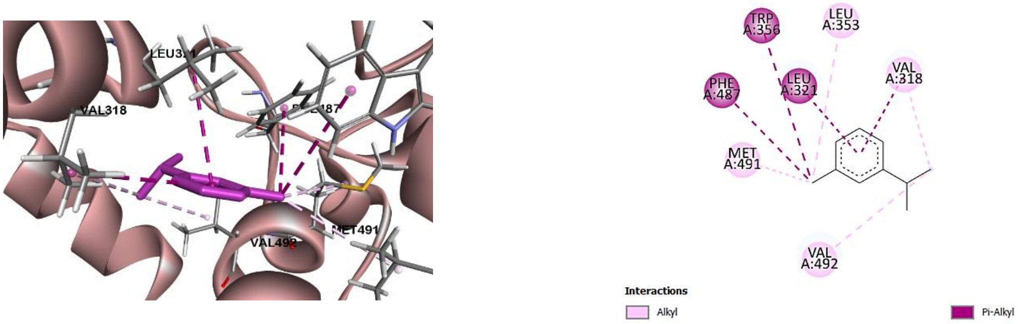

PTGS2_*trans*-Verbenol

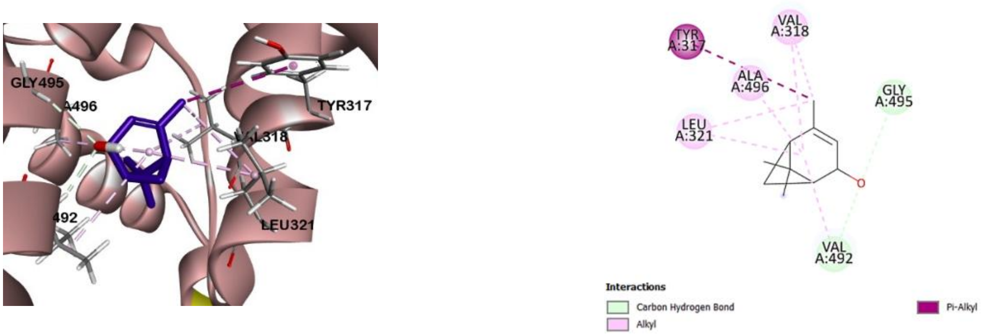

PTGS2_γ-Terpinene

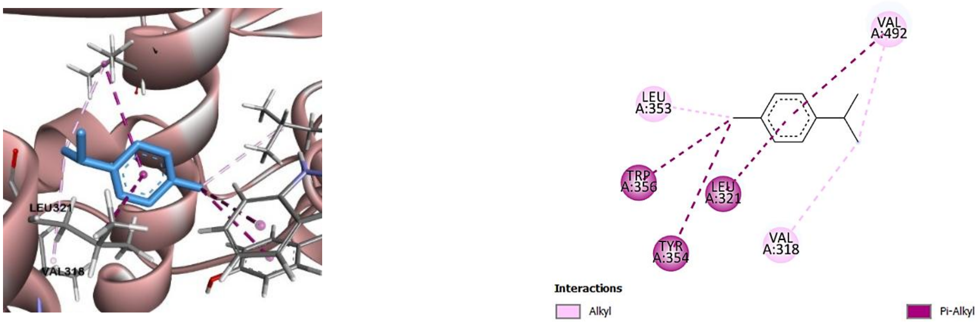

PTGS2_Fenchol

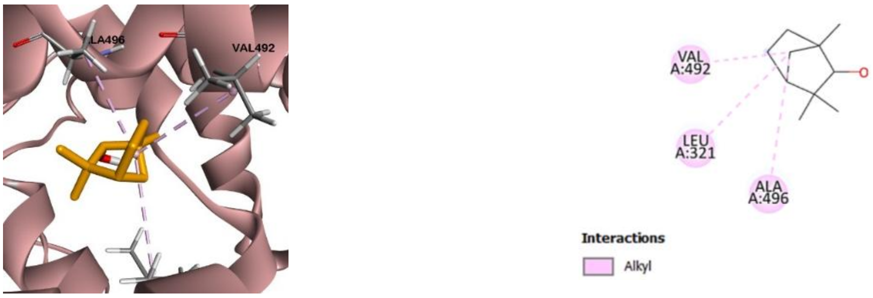

PTGS2_1R-*α*-Pinene

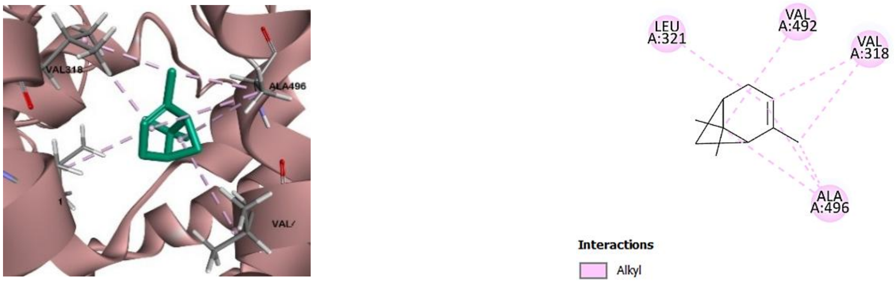

PTGS2_1S-*α*-Pinene

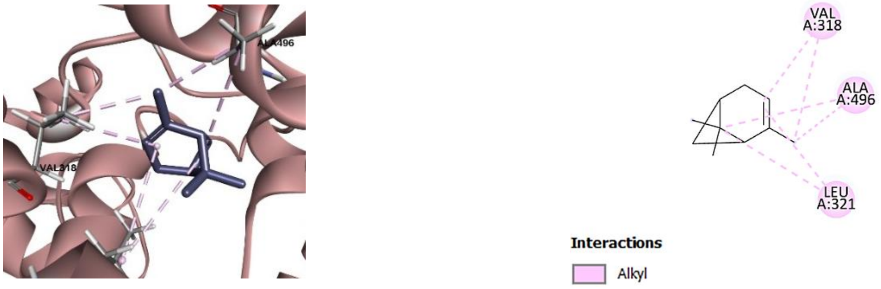

PTGS2_Terpinen-4-ol

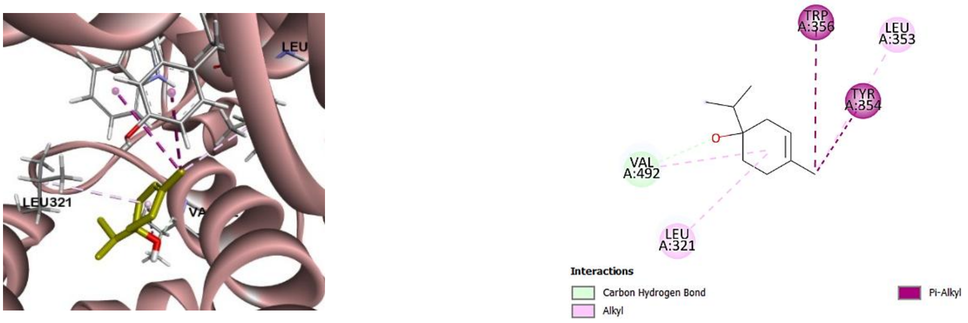

PTGS2_Isoborneol

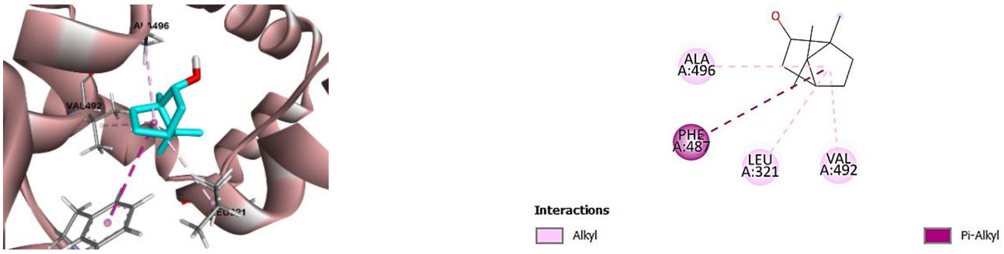

PTGS2_1,8-Cineole

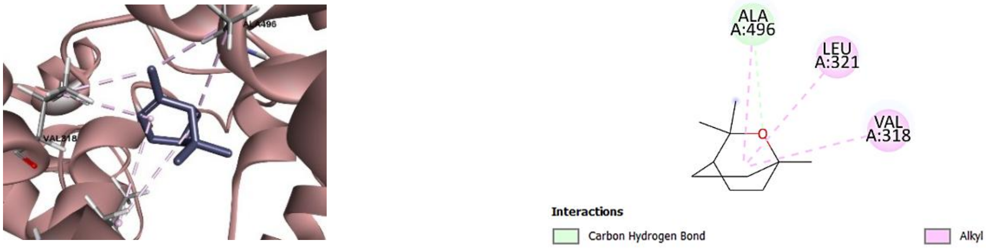

Figure S5. 3D and 2D representations of the interaction complexes of ligand-PTGS2 interactions.
