# Supplementary material for: Chemical Composition and Anti-Lung Cancer Activities of Melaleuca quinquenervia Leaf Essential Oil: Integrating Gas Chromatography–Mass Spectrometry (GC/MS) Profiling, Network Pharmacology, and Molecular Docking
Source: Pharmaceuticals (Basel). 2025 May 22;18(6):771. doi: 10.3390/ph18060771 (PMC12196179; doi:10.3390/ph18060771)
Supplement: Supplementary file 1 [file pharmaceuticals-18-00771-s001.zip › pharmaceuticals-3625015-supplementary/Revised_Supplementary_pharmaceuticals/Figure S6.pdf]

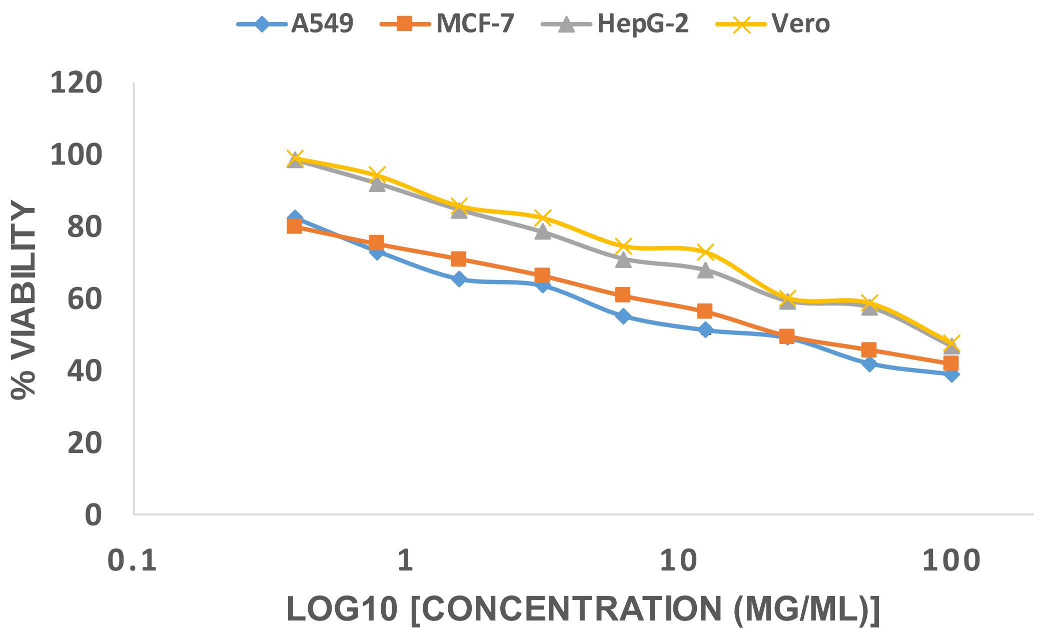

Figure S2. Dose-response curves illustrating the viability of Vero, MCF-7, HepG-2, and A-549 cell lines following exposure to various concentrations of *M. quinquenervia* leaf essential oil.
