# Supplementary material for: Chemical Composition and Anti-Lung Cancer Activities of Melaleuca quinquenervia Leaf Essential Oil: Integrating Gas Chromatography–Mass Spectrometry (GC/MS) Profiling, Network Pharmacology, and Molecular Docking
Source: Pharmaceuticals (Basel). 2025 May 22;18(6):771. doi: 10.3390/ph18060771 (PMC12196179; doi:10.3390/ph18060771)
Supplement: Supplementary file 1 [file pharmaceuticals-18-00771-s001.zip › pharmaceuticals-3625015-supplementary/Revised_Supplementary_pharmaceuticals/Table S11.docx]

**Table S1.** Target proteins, their corresponding grid coordinates, and the amino acid residues of their active sites.

| Target protein | PDB ID | Grid box coordinates | | Amino acid residues of the active site |
| --- | --- | --- | --- | --- |
|  |  | **Centers (x, y, z)** | **Sizes (x, y, z)** |  |
| ESR1 | 2OUZ | 32.2501, -1.43812, 23.1088 | 23.3876, 21.3025, 30.7697 | SER36, MET37, MET38, LEU41, THR42, LEU44, ALA45, ASP46, GLU48, LEU49, GLU75, TRP78, LEU79, LEU82, MET83, LEU86, ARG89, PHE99, GLN109, GLY110, CYS112, VAL113, GLU114, MET116, ILE119, LEU123, GLY216, MET217, HIS219, LEU220, TYR221, MRT223, LYS224, CYS225, VAL228, VAL229, PRO230, LEU231, LEU234 |
| CASP3 | 5I9B | -14.7988, -40.6385, 3.04952 | 17.5805, 17.3635, 17.9062 | SER1, GLY2, ILE3, SER4, LEU5, ASP6, ASN7, SER8, TYR9, LYS10, MET11, ASP12, GLU234, TYR236, TYR238, HIS239, LEU240 |
| PPARG | 8ATY | 6.73478, 46.8998, 79.0644 | 20.2494, 23.3903, 32.1282 | TYR22, PHE26, PRO27, LEU28, THR29, LYS30, ILE49, LEU55, GLU59, ILE62, LYS63, PHE64, LYS65, ILE67, THR68, PRO69, GLU72, GLN73, ARG80, ILE81, PHE82, GLN83, GLY84, CYS85, GLN86, PHE87, ARG88, SER89, GLU91, ALA92, GLU95, ILE96, HIS123, ILE126, TYR127MET129, LEU130, SER132, LEU133, VAL139, LEU140, ILE141, SER142, GLU143, GLY144, MET148, PHE163, MET164, LYS167, LEU184, HIS249, LEU253, LEU269, TYR273 |
| PTGS2 | 5F19 | 25.9892, 42.8975, 54.3703 | 48.5574, 45.8497, 50.8006 | LYS1, ASN2, PRO3, CYS4, HIS7, PRO8, CYS9, GLN10, ASN11, ARG12, GLY13, VAL14, CYS15, MET16, SER17, PHE20, THR28, ARG29, THR30, GLY31, PHE32, THR44. LYS47, LEU48, LYS51, PRO52, PRO54, VAL57, HIS58, LEU61, ILE81, MET82, TYR84, VAL85, THR87, SER88, ARG89, SER90, HIS91, LEU92, ILE93, ASP94, SER95, PRO96, PRO97, THR98, TYR99, ALA101, ASP102, TYR103, GLY104, TYR105, LYS106, TRP108, GLU109, PHE111, SER112, ASN113, LEU114, SER115, THR118, ARG119, ALA120, LEU121, PRO122, PRO123, VAL124, PRO125, ASP126, ASP127, GLN161, PHE174, THR175, PHE178, LEU193, GLY194, HIS195, GLY196, VAL197, ASP198, ASN200, GLY204, GLU205, THR206, LEU207, GLN210, GLU291, TRP292, GLY293, GLU295, GLN296, GLN299, THR300, ARG302, LEU303, VAL313, ILE314, TYR317, VAL318, LEU321, SER322, TYR324, PHE326, LEU328, LEU335, PHE336, ASN337, LYS338,GLN339, PHE340, GLN341, TYR342, GLN343, ASN344, ARG345, ILE346, ALA347, PHE350, LEU353, TYR354, TRP356, LYS428, GLN430, GLU434, LYS437, ARG438, PHE439, MET440, LEU441, ARG482, ALA485, ILE486, PHE487, MET491, VAL492, GLU493, GLY495, ALA496, PHE498, LEU500, LYS501, GLY502, LEU503, GLY505, ASN506, VAL507, SER510, PRO511, ALA512, LYS515, SER517, THR518, GLY520, |
